# Phytochemical diversity and therapeutic potential of *Verbesina* metabolites: a critical review of sesquiterpene lactones and translational feasibility

**DOI:** 10.3389/fphar.2026.1899545

**Published:** 2026-08-28

**Authors:** Karla Irazu Ventura-Hernández, Tushar Janardan Pawar, Fernando Rafael Ramos-Morales, Shrikant S. Nilewar, Carlos Alberto López-Rosas, Fabiola Hernández-Rosas

**Affiliations:** 1 Instituto de Química Aplicada, Universidad Veracruzana, Xalapa, Veracruz, Mexico; 2 Centro de Investigación, Universidad Anahuac Querétaro, El Marqués, Querétaro, Mexico; 3 Escuela de Ingeniería Química, Universidad Anahuac Querétaro, El Marqués, Querétaro, Mexico; 4 Department of Pharmaceutical Chemistry, Maliba Pharmacy College, Uka Tarsadia University, Bardoli, Gujarat, India; 5 Facultad de Química Farmacéutica Biológica, Universidad Veracruzana, Xalapa, Mexico; 6 Escuela de Ingeniería Biomédica, División de Ingeniería, Universidad Anáhuac Querétaro, El Marqués, Querétaro, Mexico; 7 Facultad de Química, Universidad Autónoma de Querétaro, Santiago de Querétaro, Querétaro, Mexico

**Keywords:** antimicrobial resistance, cytotoxicity, ethnobotany, sesquiterpene lactones, therapeutic index, *Verbesina*

## Abstract

The global health crisis driven by Antimicrobial Resistance (AMR) necessitates a pivot toward novel therapeutic agents, yet the Asteraceae genus *Verbesina* remains significantly under-characterized. Despite comprising over 300 species, early assessments indicated that only approximately 10% had been biologically investigated. This review provides a critical synthesis of the pharmacological landscape of *Verbesina*, focusing on the structural duality of its major specialized metabolites, the Sesquiterpene Lactones (SLs). The review systematically analyzes reported *in vitro* data, emphasizing high-potency scaffolds such as 6β-cinnamoyloxy-3β,4α-dihydroxyeudesmane (MIC = 3.9 μg/mL) and Verbesindiol (IC_50_ = 4.0 ± 0.6 μM) against prostate cancer cells. A core focus is placed on the Selectivity Index (SI = IC_50_/MIC); to address the translational challenge of non-selective toxicity. Notably, an Arbusculin derivative demonstrates a superior SI of up to 88.2, significantly outperforming standard drugs like meglumine antimoniate (SI = 40.0). Furthermore, the review evaluates the molecular mechanisms of action, such as the role of the α-methylene-γ-lactone moiety in alkylating cellular targets, while distinguishing structure-specific effects like mitochondrial uncoupling and respiratory chain inhibition. By bridging ethnopharmacology with modern mechanistic and quantitative data, this review critically assesses the translational potential of *Verbesina* metabolites, exposes current methodological limitations in the literature, and highlights clear directions for bioassay-guided isolation and chemical optimization as next-generation anti-resistance scaffolds.

## 1 Introduction

The rapid and relentless emergence of multidrug-resistant (MDR) microbial pathogens represents one of the most pressing and complex global health threats of the 21st century (Grichar and Sestak, 1998). The World Health Organization (WHO) categorized Antimicrobial Resistance (AMR) as a critical priority, forecasting a dire scenario where common infections and minor injuries could once again become life-threatening (Inderjit et al., 2000; Panero et al., 1999). This crisis is exacerbated by the overuse of existing antibiotics and a severe slowdown in the discovery pipeline for new therapeutic agents with novel mechanisms of action (Chappelka et al., 2003). Consequently, the scientific community faces an imperative to explore nature’s vast chemical library for new scaffolds capable of overcoming established bacterial defense mechanisms. Historically, natural products have been the undisputed bedrock of modern pharmacology, with approximately 60% of current antibacterial and anticancer agents derived directly from natural sources or semi-synthetic modifications thereof (Grichar et al., 2012). These biologically evolved molecules possess unparalleled structural complexity, allowing them to interact with complex biological targets in unique ways.

Among the plant families recognized for their phytochemical richness and pharmacological significance, the Asteraceae (formerly Compositae) stands out (Evans and Borowicz, 2013). This family is the largest in Mexico, a nation designated as “Megadiverse,” which houses a high concentration of unique chemical diversity and is considered an “evolutionarily active zone” dueting to an accelerated diversification rate within the Asteroideae subfamily (Carmona-Chit et al., 2016; Sharma et al., 2018). The chemical diversity within Asteraceae is staggering, yielding a wide array of specialized metabolites, including polyacetylenes, flavonoids, alkaloids, and notably, various classes of terpenoids (Evans and Borowicz, 2013; Kaur et al., 2021; Rivera et al., 2021).

Within this prolific family, the Sesquiterpene Lactones (SLs) form a pivotal class of secondary metabolites, characterized by an isoprenoid structure containing 15 carbon atoms and a lactone function (Kaur et al., 2022). These SLs are structurally classified into several major skeletal groups, including germacranolides, guaianolides, and eudesmanolides (Kaur et al., 2021). The SLs are of paramount interest to medicinal chemists due to the consistent presence of a highly reactive functional group: the α-methylene-γ-lactone moiety (Kaur et al., 2022). This structural feature is widely regarded as the primary pharmacophore responsible for their profound biological activities. The unsaturated lactone ring acts as a non-specific alkylating agent, undergoing a Michael-type addition reaction with biological nucleophiles, particularly the sulfhydryl (-SH) groups found in cysteine residues of proteins and peptides like glutathione (Kaur et al., 2022) This alkylation mechanism provides a clear, unifying hypothesis for their therapeutic potential across both microbial and proliferative targets.

The genus Verbesina itself encompasses over 300 species, with the greatest diversity concentrated in Mexico and the southwestern USA (Velasco-Ramírez et al., 2022a). Phylogenetic analysis confirms *Verbesina* as a distinct lineage within the Heliantheae alliance (Carmona-Chit et al., 2016; Mehal et al., 2023). Reinforcing its status as a reliable repository of potent specialized metabolites. This chemical potency is not accidental; the genus’s bioactivity is fundamentally linked to its ecological role as an aggressive chemical warrior (Tomasello et al., 2024; Wu et al., 2024). Species like *Verbesina encelioides* (Cav.) Benth. and Hook.f. ex A.Gray [Asteraceae] are notorious invasive weeds globally (Mehal et al., 2023), capable of quickly dominating ecosystems due to potent allelopathic properties, which involve the competitive release of phytotoxic chemicals (Hernández-Pérez et al., 2025; Verma et al., 2025; Wu et al., 2024). This high ecological competence and survival, even in contaminated environments, underscore the necessity and effectiveness of its underlying chemical arsenal (Burkey et al., 2006; Carmona-Chit et al., 2016; Velasco-Ramírez et al., 2022a).

Phytochemical investigation has consistently identified characteristic metabolites: the dominant SLs are primarily of the eudesmanolide type, a scaffold that accounts for almost half of all natural eudesmanolides found in the Asteraceae family (Velasco-Ramírez et al., 2022b; Wu et al., 2024). These compounds are often isolated as cinnamoyloxy eudesmane conjugates, a defining chemical signature of the genus. Historically, the initial isolation and structural elucidation of these cinnamoyloxy esters from species like *Verbesina virginica* L. [Asteraceae] established this complex scaffold as the functional heart of *Verbesina*’s chemistry (Herz and Kumar, 1981).

The genus’s therapeutic relevance is deeply rooted in Mexican ethnobotany, where various species are integrated into local practices for treating ailments related to inflammation, gastrointestinal distress, and topical infections (Mora et al., 2013). For instance, *Verbesina crocata* (Cav.) Less. [Asteraceae] is traditionally used for wound healing and antidiabetic activity (Bohlmann et al., 1982; Albalawi et al., 2015).

Beyond anti-infective use, the therapeutic scope is rapidly expanding, with recent studies validating the plant’s dual function as a valuable resource for sustainable agriculture acting as potent plant biostimulants that promote crop yield (Grichar et al., 2012; Hernández-Pérez et al., 2025; Velasco-Ramírez et al., 2022a) and neuroprotection showing efficacy against Alzheimer’s disease models (Verma et al., 2025).

However, the powerful, non-selective chemical reactivity inherent in the α-methylene-γ-lactone group, the very mechanism responsible for efficacy presents a critical translational challenge: the inevitable duality of efficacy and toxicity (Kaur et al., 2022). The same alkylating mechanism that deactivates bacterial enzymes can interfere with vital homeostatic processes in non-target, healthy mammalian cells (Bohlmann et al., 1982; Farshori, 2021; Albalawi et al., 2015). This potential non-selective toxicity is vividly underscored by the genus’s history, as many species contain highly toxic compounds like the guanidine alkaloid, galegine, which is fatal to livestock (Oelrichs et al., 1981; Sharma et al., 2018).

Therefore, a purely potent finding in an antimicrobial assay (low Minimum Inhibitory Concentration, MIC) or a cytotoxicity assay (low IC_50_) is insufficient. Success depends entirely on the compound’s ability to preferentially target pathogenic or diseased cells over healthy ones. This necessitates a critical quantitative comparison of these two parameters by calculating the Selectivity Index (SI = IC_50_/MIC) (Indrayanto et al., 2021). This metric is essential for advancing natural product research toward clinical application, providing the necessary assurance that potent compounds are also safe.

**FIGURE 1:**
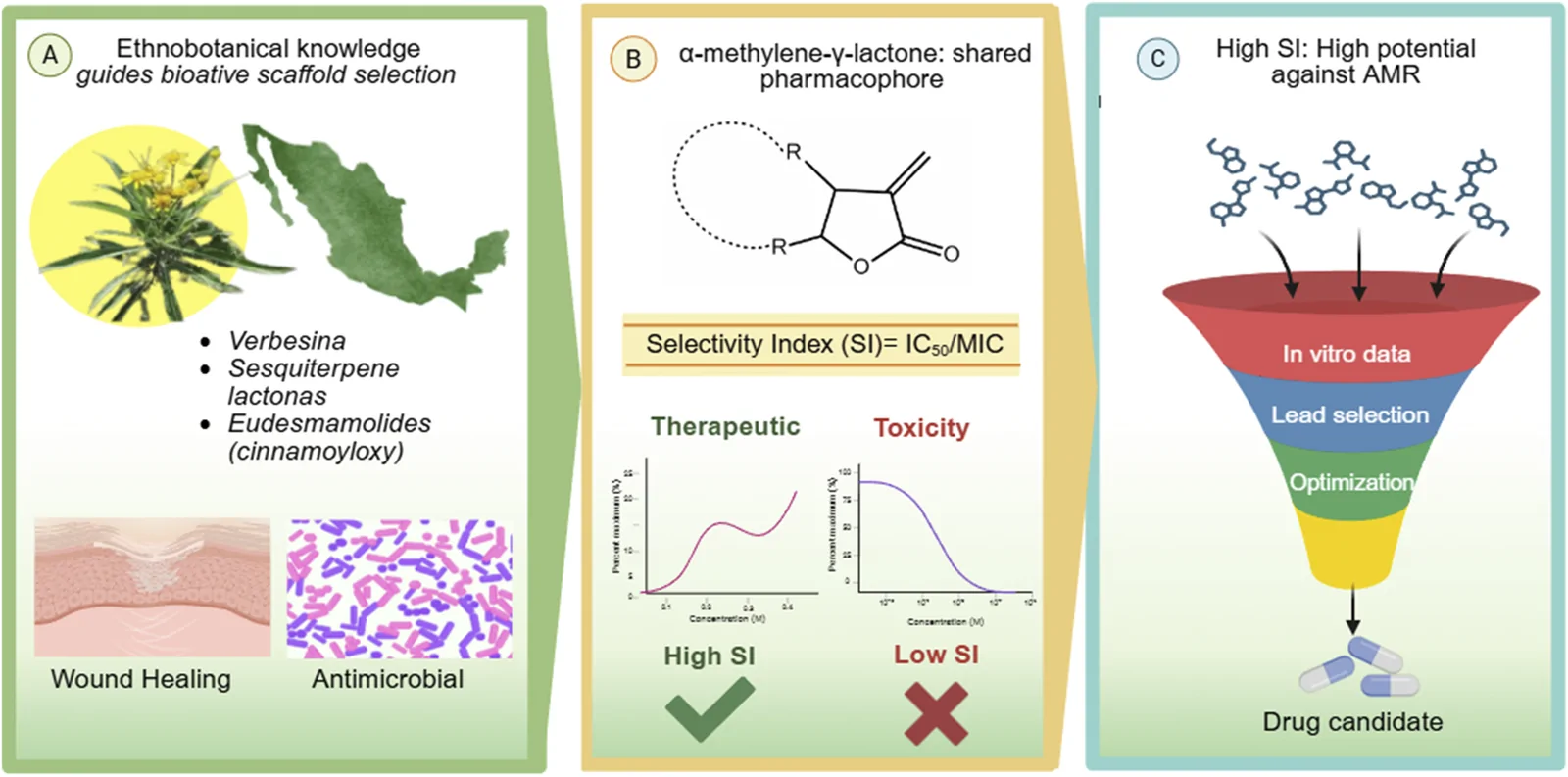
The Duality Challenge and Translational Road Map. Conceptual framework for the identification of *Verbesina*-derived bioactive compounds. Ethnobotanical knowledge supports scaffold selection, especially sesquiterpene lactones with wound-healing and antimicrobial potential **(A)**. The α-methylene-γ-lactone motif is highlighted as a common pharmacophore, and compound prioritization is based on the selectivity index (SI = IC50/MIC), balancing therapeutic activity and toxicity **(B)**. Compounds with high SI and promising activity against antimicrobial resistance (AMR) advance through *in vitro* screening, lead selection, and optimization toward drug candidate development **(C)**.

Despite compelling ethnobotanical and preliminary pharmacological data, a critical gap exists in the synthesized literature: the absence of a focused, comparative analysis that quantifies the SI for its constituent Sesquiterpene Lactones. This review aims to address this gap by providing a comprehensive, mechanistic, and quantitative analysis of the *Verbesina* genus’s potential as a source of anti-resistance therapeutics. We will critically evaluate the literature regarding the cytotoxicity (IC_50_) and antimicrobial efficacy (MIC) of its specialized metabolites, synthesizing all available *in vitro* data to calculate and interpret the Therapeutic Index for key scaffolds. By focusing on the structural relationships, dual biological activities, and selective toxicity profiles, this review will delineate the most promising scaffolds for further chemical development, ultimately validating the translational potential of this rich Mexican medicinal resource in the fight against AMR (Figure 1). The structural and pharmacological focus of this review is deliberately centered on the sesquiterpene lactone (SL) class, as these compounds represent the primary chemotaxonomic markers of *Verbesina* and serve as the fundamental molecular drivers of the genus’s characteristic efficacy-toxicity duality. Other co-occurring metabolite classes, including flavonoids, triterpenoids, phenolic acids, and alkaloids, are systematically evaluated through the specific lens of how they structurally modulate host safety boundaries, exhibit toxicological synergy, or explain historical discrepancies between traditional aqueous preparations and organic extracts.

## 2 Materials and methods

A comprehensive literature search was conducted in Scopus, Web of Science Core Collection, SciFinder, and Google Scholar from database inception to 17 December 2025, without restrictions on publication year, language, geographical origin, or study design. The search combined the genus name *Verbesina*, the accepted scientific names of the species, and their taxonomic synonyms with terms related to phytochemistry, specialized metabolites, sesquiterpene lactones, eudesmanes, ethnopharmacology, biological activity, toxicity, antimicrobial activity, cytotoxicity, and selectivity. The general Boolean strategy was (“Verbesina” OR accepted species name OR taxonomic synonym) AND (“phytochem*” OR “specialized metabolite*” OR “sesquiterpene lactone*” OR “eudesmane*” OR “ethnopharmacolog*” OR “antimicrobial” OR “antibacterial” OR “antifungal” OR “antiprotozoal” OR “cytotoxic*” OR “toxicity” OR “selectivity index” OR “therapeutic index”). The syntax was adapted to the specific requirements of each database, and additional searches were conducted separately for each accepted species name and its documented synonyms to retrieve older studies indexed under historical botanical names. Reference lists of retrieved articles and relevant reviews were also screened manually to identify additional publications. Overlapping records retrieved from different databases were identified during the screening process, and titles, abstracts, and full texts were examined to determine their relevance to the scope of the review. Publications were included when they provided taxonomic, phytochemical, ethnopharmacological, pharmacological, toxicological, or biological information directly attributable to an authenticated *Verbesina* species, extract, fraction, essential oil, or isolated metabolite. Records were excluded when *Verbesina* was mentioned only incidentally, when species-specific information was unavailable, or when the reported data could not be attributed to a defined botanical material or metabolite. Botanical names were verified using Plants of the World Online and Medicinal Plant Names Services, and the accepted name, botanical authority, family, and available synonyms were recorded for each taxon. Historical names used in the original publications were cross-referenced with the currently accepted nomenclature. Relevant information was extracted into a standardized matrix covering taxonomic identity, voucher specimen, plant part, extraction procedure, chemical characterization, analytical methods, major metabolites, biological activity, quantitative pharmacological parameters, principal findings, and methodological limitations. Primary experimental studies were prioritized for the analysis of antimicrobial activity, cytotoxicity, and selectivity, and studies involving botanical extracts, fractions, or essential oils were assessed according to ConPhyMP recommendations.

## 3 Ethnopharmacology and phytochemical diversity of the *Verbesina* Genus

The rational investigation of the *Verbesina* genus, which remains the largest genus within the Heliantheae tribe, is firmly anchored in its definitive chemotaxonomic and phylogenetic placement. Molecular studies confirm that *Verbesina* belongs to the subtribe Verbesininae, a distinct and ancient lineage nestled within the core Heliantheae clade (Panero et al., 1999). This confirmed taxonomic position refines the focus for all chemical investigation, supporting the established consistency of the genus’s potent eudesmane SLs as strong chemotaxonomic markers (Tomasello et al., 2024). This structural link is so vital that contemporary research has focused on assembling the complete chloroplast genome (*plastome*) for *Verbesina alternifolia* (L.) Britton ex Kearney [Asteraceae], providing a robust genomic anchor necessary for targeted bioassay-guided isolation and standardization across genetically diverse populations (Tomasello et al., 2024).

### 3.1 Ethnobotanical and ecological drivers of therapeutic duality and translational feasibility

The foundational relevance of the *Verbesina* genus is established through its chemotaxonomic and phylogenetic placement (Panero et al., 1999). Molecular data firmly place *Verbesina* within a distinct clade, often recognized as the revised subtribe Verbesininae (Panero et al., 1999) nestled in the larger Heliantheae alliance clade, which accounts for approximately 26% of all species in the *Asteraceae* family (Rivera et al., 2021). This refined circumscription, separating *Verbesina* from other common allies, acts as a crucial DNA-based chemotaxonomic anchor that directs and refines future bioassay-guided isolation work (Panero et al., 1999). Geographically, the genus is highly prolific, encompassing over 300 species with the greatest diversity concentrated in Mexico and the southwestern USA (Rivera et al., 2021). The widespread and robust distribution of *Verbesina* across central Mexican flora, including regions of high diversity like the Bajío, underscores its pharmacological importance (Villaseñor and Ortiz, 2012). Furthermore, Mexico is positioned as an “evolutionarily active zone” for this lineage, characterized by high speciation rates within the *Asteroideae* subfamily, which accelerates the diversification of the genus’s potent specialized metabolites (Rivera et al., 2021). The specific niche preference, such as *V. virginica* strictly inhabiting areas under the canopy, suggests that the plant’s metabolic investment in synthesizing a robust chemical arsenal, including SLs, necessitates a sheltered microclimate to maximize chemical production (Van Auken and Leonard, 2016). The extensive geographical distribution and success are also supported by studies that investigate cellular mechanics, including cytological analysis of *V. encelioides*, which has shown genetic variability often linked to the plant’s aggressive ecological adaptation and the synthesis of secondary metabolites (Kaur et al., 2015).

The pharmacological investigation of *Verbesina* is directly guided by its established history in Mexican traditional medicine (García-Bores et al., 2020), which involves a broader cultural reliance on potent, locally sourced medicinal flora throughout the nation (Domínguez-Barradas et al., 2015). For instance, *Verbesina persicifolia* DC [Asteraceae]: or ‘Huichín,' is a popular folk remedy confirmed for anti-diabetic activity (Perez et al., 1996) and is used for ailments including inflammatory, gastrointestinal, and anti-infective complaints like wounds that resist healing (Dalla Via et al., 2015; Martínez Alfaro et al., 1995). Similarly, the endemic *V. crocata* is traditionally applied topically for wounds and burns (García-Bores et al., 2020), and *Verbesina sphaerocephala* A. Gray [Asteraceae] is locally applied for treating gangrene and venous ulcers (Rodríguez-Valdovinos et al., 2021). Scientific reports have successfully confirmed the efficacy for three traditional uses: wound healing, antidiabetic, and diuretic activity (Xu et al., 2010). Most recently, *V. persicifolia* has been validated for applications related to the central nervous system, such as anticonvulsant activity (López-Rosas et al., 2025) broadening its therapeutic scope beyond anti-infective use. The traditional use of these compounds for infections and inflammation provides the essential ethnopharmacological rationale for investigating its modern anti-AMR properties (Martínez Alfaro et al., 1995; Mora et al., 2013). Furthermore, the anti-infective potential extends beyond the Mexican context, with species like the South American *Verbesina macrophylla* (Cass.) S.F.Blake [Asteraceae] used traditionally for bacterial and fungal infections of the urinary and respiratory tracts (de Veras et al., 2021).

The plant’s inherent bioactivity is fundamentally linked to its ecological success as an aggressive chemical warrior (Al-Sodany et al., 2018; Essinger et al., 2020). The high ecological competence of species like *V. encelioides* is evidenced by its recognition as a weed (Grichar and Sestak, 1998) and its aggressive colonization ability is enhanced by strong allelopathic properties (Al-Sodany et al., 2018). Aggressive success is intrinsically linked to its ability to adapt to diverse and often disturbed habitats, with species like *V. alternifolia* surviving in fire-affected systems, highlighting the environmental pressures that drive the constant synthesis and selection of potent specialized metabolites (Van Nuland et al., 2013). This chemical defense system includes not only defense against herbivory but also compounds vital for pollinator attraction (Van Nuland et al., 2013). Rather than depending on generalized structural defenses, the potent chemical arsenal of the genus is physically manufactured and housed in specialized defensive structures common across its tribe (Aschenbrenner et al., 2013). Anatomical studies confirm the ubiquitous presence of Linear Glandular Trichomes (LGTs) on the plant surfaces that are metabolically active and primarily accumulate terpenoids and other specialized metabolites (Aschenbrenner et al., 2013). This rich chemical arsenal extends beyond SLs to include a robust mixture of triterpenoids and phytosterols like β-Amyrin and Lupeol (Sultana et al., 2018), which may possess anti-diabetes and antidyslipidemic properties, suggesting the non-SL fraction provides synergistic or mitigating activities important for the overall therapeutic profile (Sultana et al., 2018). Finally, the selection of *Verbesina* for drug discovery is justified by its high degree of genetic and ecological resilience, confirmed by molecular studies showing a significant genetic distance from cultivated *Asteraceae* and the capacity for producing diverse and potent specialized metabolites to survive in competitive environments (Bonchev and Vassilevska-Ivanova, 2020).

However, translating this aggressive chemical defense network into safe clinical interventions requires evaluating the strict boundaries between ecological chemical warfare (non-specific toxicity) and clinical selectivity. This structural duality necessitates that these ethnobotanically targeted anti-infective agents are systematically screened using unified, paired quantitative metrics (MIC and IC_50_) to determine a reliable Selectivity Index (SI) threshold for downstream drug development.

### 3.2 The core chemical landscape: sesquiterpene lactones

#### 3.2.1 Eudesmane sesquiterpene lactones

**FIGURE 2:**
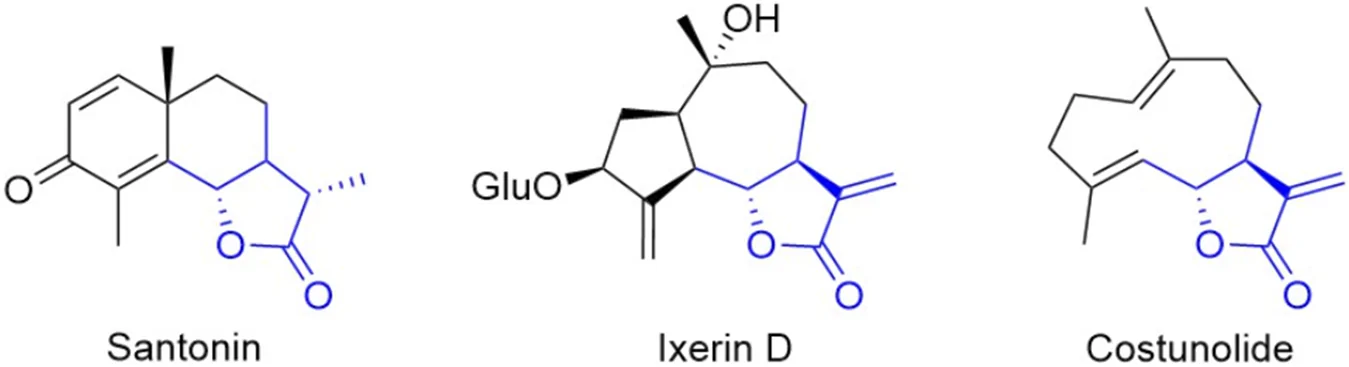
Core SL Skeletons and Pharmacophore. Santonin, ixerin D and costunolide illustrate the structural diversity of sesquiterpene lactones while preserving the characteristic lactone ring. The highlighted α-methylene-γ-lactone moiety represents a pharmacophore associated with the biological activity of this metabolite class.

The chemical identity of the *Verbesina* genus is structurally defined by the Sesquiterpenoid class, which are overwhelmingly the most abundant specialized metabolites, constituting approximately 79% of all Terpenoids reported in the genus (Mora et al., 2013). This rich biosynthetic output is concentrated around the bicyclic C_15_ eudesmane skeleton (Wu et al., 2024), a scaffold recognized for its wide range of biological activities, including anti-tumor, antifungal, and antibacterial properties (Figure 2) (Kaur et al., 2021; Wu et al., 2006). The historical foundation began with seminal research on the roots of *V. virginica* in 1961, which reported the isolation of α- and β-Verbesinol derivatives (Gardner et al., 1961). These compounds possessed the C15 sesquiterpene framework with a *ci*s-decalin (eudesmane) ring system. Crucially, this study determined that the compounds existed naturally as isomeric sesquiterpene esters which, upon degradation, yielded *p*-hydroxycinnamic acid (Gardner et al., 1961). This established the existence of the defining cinnamoyloxy eudesmane scaffold, a structure that links a lipophilic terpenoid body to a phenolic acid (the cinnamoyl moiety) confirming the characteristic chemical structure that governs the genus’s potent bioactivity (Bohlmann and Lonitz, 1978; Gardner et al., 1961). This overwhelming structural prevalence means that eudesmane cinnamates alone account for over a quarter (26.5%) of all reported *Verbesina* compounds, firmly validating the structural focus of this review (Mora et al., 2013). From an SI optimization perspective, this cinnamate ester serves as a non-covalent structural anchor that fixes the molecule into target binding pockets, pre-aligning the highly reactive α-methylene-γ-lactone warhead for irreversible alkylation. Representative sesquiterpenoid scaffolds and their structural characteristics are summarized in Table 1.

**TABLE 1:** Structural diversity of key sesquiterpenoids (SLs and core analogues) from the verbesina genus.

Compound name (skeleton)	Structural descriptor	Source species	Biological role
Verbesindiol (Eudesmane - Diol)	Parent core; 4*R*,5*S*,6*R*,7*S*,10*R* configuration; Structural standard	*V. virginica, V. eggersii*	Potent against prostate cancer cells
CDE (Eudesmane Cinnamate)	4β-cinnamoyloxy, 1β,3α-dihydroxyeudesm-7,8-ene; Alkylating pharmacophore	*V. persicifolia*	Mild mitochondrial uncoupler Patented for obesity/diabetes/cancer treatment
Arbusculin derivative (Eudesmane Lactone)	(1*R*,4*S*,4a*S*,7*R*)-1-methyl-7-(prop-1-en-2-yl)octahydro-2*H*-1,4a-(epoxymethano)naphthalen-4-yl acetate	*V. lanata*	Against the protozoan *L. major*
Rupestrol (Pentahydroxy Eudesmane)	Highly oxygenated enantio-eudesmane with C-9 oxygenation; isolated as cinnamate esters	*V. rupestris*	Confirms complex stereochemistry/chemotaxonomic links
Rupestrinol (Enantio-Eudesmane)	Epimeric sesquiterpene isolated as cinnamate esters	*V. rupestris*	Part of the complex enantio-eudesmane structural series
Eudesmane Esters (Eudesmane Cinnamate)	Phenolic acid (cinnamate/coumarate) conjugated eudesmanes	*V. virginica*	Upholds the chemical signature of SLs conjugated with phenolic acids
SL Acetylated Derivatives (Eudesmane/Germacrane)	Compounds with acetate substituents	*V. persicifolia*	Acetylation shifts cytotoxicity mechanism to Complex II inhibition (Mitochondrial)
Germacrene-Cinnamate	6β-cinnamoyloxy-1β-hydroxy-10α-methoxy-3-oxo-germacra-4,5*Z*-ene	*V. negrensis*	Antibacterial against *S. aureus*
Zempoaline C (Elemanolide)	Elemanolide skeleton with fused α,β-unsaturated-γ-lactone moiety	*V. seatonii*	Deploys alkylating pharmacophore via diverse skeletal type
Verocephol (Amorphane Lactol)	Unique γ-lactol; first reported amorphane skeleton	*V. sphaerocephala*	Marks high biosynthetic diversity (C_15_ precursors)
Cadinenes (Sesquiterpenoid)	New Cadinene-type SLs confirmed by X-ray	*V. sphaerocephala*	Structural diversity increases selective lead probability
Parthenin Analog (SL)	SLs induce cytotoxicity via Michael addition to -SH groups	*(General SL mechanism)*	α-methylene-γ-lactone is the alkylating pharmacophore
*V. macrophylla* EO (Sesquiterpenes)	Essential Oil dominated by C_15_ Sesquiterpenes (Germacrene D)	*V. macrophylla*	Antimicrobial/anti-inflammatory action with very low host toxicity
Anthelmintic Action (Sesquiterpenes/Phenolics)	Essential oil shows 100% adulticidal activity against Habronema muscae	*V. alternifolia*	Causes degenerative changes to parasite morphology (SEM confirmed)

The consistency of this chemical signature is reinforced by systematic investigations of South American *Verbesina* species (including *Verbesina glabrata* Hook. and Arn. [Asteraceae], *V. macrophylla*, and *Verbesina luetzelburgii* Mattf. [Asteraceae]), which confirmed consistent chemical dominance with all species affording eudesmane or germacrane derivatives, nearly all bearing a cinnamate or related ester residue (Bohlmann et al., 1980). Further supporting this claim, a phytochemical investigation into the aerial parts of *Verbesina turbacensis* Kunth [Asteraceae] (a species of ethnobotanical importance) successfully isolated four eudesmane derivatives (three of which were novel), further reinforcing the ubiquitous nature of this C15 bicyclic scaffold (Amaro-Luis et al., 2002).

The early chemical investigation of the genus was often challenged by the complexity and high oxygenation of these terpenoids. For instance, the structural elucidation of Rupestrol from *Verbesina rupestris* S.F.Blake [Asteraceae] (Jamaica) detailed a pentahydroxy sesquiterpene of the eudesmane type, featuring hydroxyl groups at C-4, C-6, and C-14, alongside secondary hydroxyls at C-1 and C-9. Chemical reactivity tests, such as the inertness of Rupestrol cinnamate to periodic acid, were required to definitively eliminate C-3 as a site for a secondary alcohol, supporting the locations at C-1 and C-9 (Box et al., 1971). Further complexity was revealed in *V. rupestris* through the isolation of enantio-eudesmane sesquiterpenes, mirror-image isomers of the common eudesmane structure including Rupestrinol and γ-Chaenocephalol, both isolated as their cinnamate esters. The isolation of these eudesmane sesquiterpenes provided the necessary chemotaxonomic evidence to support the botanical reclassification of *Chaenocephalus rupestris* to *V. rupestris* (Box et al., 1977).

Establishing a reliable Structure-Activity Relationship (SAR) requires an unequivocal definition of the molecule’s C_15_ scaffold, particularly its stereochemistry (Ramseyer et al., 2017). This was achieved through a series of meticulous structural confirmations. The *p*-coumaryl ester of the new sesquiterpenediol, Verbesindiol, was isolated from the herbaceous parts of *V. virginica*, and alkaline hydrolysis confirmed it was an Eudesmane derivative with hydroxyl groups located at the tertiary C-4 position and a second at C-6. The relative and absolute configuration was definitively established (*4R*,*5S*,*6R*,*7S*,*10R*) using ^1^H-NMR, ^13^C-NMR, and Circular Dichroism (CD) spectroscopy (Herz & Kumar, 1981). This structural work culminated in the X-ray crystallographic analysis of the parent diol, which provided the absolute configuration and definitively established the Verbesindiol core as a 4α,6β-dihydroxyeudesmane with a *cis*-fused decalin ring system. This structure, characterized by the specific orientation of the hydroxyls at C-4 and C-6, serves as the absolute standard for correlating cinnamoyloxy eudesmane scaffolds with their observed biological activities (Herz et al., 1982). A detailed reinvestigation of *Verbesina eggersii* Hieron. [Asteraceae] consistently confirmed the ubiquity of Verbesindiol derivatives across the genus, noting that this species is rich in cinnamoyloxy eudesmane SLs and yielded further derivatives, including 15-hydroxy derivatives, a rearranged eudesmane acid, and a benzofuran derivative, highlighting the propensity of the Asteraceae to produce structurally diverse scaffolds that originate from the same basic terpenoid precursors (Banerjee et al., 1985).

The necessity for definitive characterization is further demonstrated by studies on Mexican endemic species. The aerial part of *Verbesina virgata* Cav. [Asteraceae], for example, afforded two new C-4 cinnamates, including 4β-cinnamoyloxy-1β,2α-dihydroxyeudesm-7-en, whose structure and stereochemistry were unambiguously determined by X-ray diffraction analysis. This analysis confirmed the *cis*-ring junction and the equatorial orientation of the C-1 hydroxyl group. The study also confirmed the presence of vicinal hydroxyl groups through periodate cleavage, demonstrating that these natural molecules possess chemical functionality capable of undergoing specific reactions with biological targets (Martinez et al., 1983). The ubiquitous presence of these Eudesmane cinnamates is further confirmed by their isolation from *Verbesina sordescens* DC. [Asteraceae], which afforded two new eudesmane cinnamates alongside a typical *Verbesina* blend of terpenoids and polyacetylenes (Bohlmann et al., 1982). The ubiquitous nature of the eudesmane skeleton in the genus continues to be proven, as an investigation into *V. virginica* yielded two new cinnamoyl-esterified eudesmane compounds, both featuring the core eudesmane ring system modified by substitution with hydroxyl groups at C-1 and C-4. Crucially, one compound was esterified with *p*-coumaric acid and the other with cinnamic acid, reinforcing the defining chemical characteristic of the genus: SLs conjugated with phenolic acids (Xu et al., 2010) The ongoing investigation continues to define the absolute configuration of key eudesmane derivatives from species like *V. turbacensis* using single-crystal X-ray crystallographic analysis, reinforcing the reliance on advanced structural data to reliably map a compound’s efficacy to its three-dimensional shape (Bruno-Colmenárez et al., 2010).

The importance of stereochemical precision for reliable SAR is highlighted by findings regarding the eudesmanetriol mono-cinnamate isolated from *Verbesina persicifolia*. This compound, identified as 4,10-dimethyl-7-isopropyl-*trans*-decalin-1,3,4-triol-7-ene-4-*trans* cinnamate, revealed a correction to the configuration of its C-4 asymmetric center compared to previous assignments in other *Verbesina* species, confirming the *trans*-decalin eudesmane backbone in certain highly active lead molecules (Cadanedo et al., 2015). A core example of the characteristic scaffold is 4β-cinnamoyloxy, 1β,3α-dihydroxyeudesm-7,8-ene, isolated from the hexane extract of *V. persicifolia* (Dalla Via et al., 2015). This eudesmane derivative is pivotal because its core scaffold, as shown through studies on the closely related CDE, can exert profound biological effects not solely through direct toxicity but through the modulation of fundamental cellular processes. For instance, the eudesmane skeleton has been shown to act as a natural mild uncoupler in liver mitochondria (Dalla Via et al., 2014). This critical evidence underscores the necessity of rigorously quantifying the IC_50_ (cytotoxicity) and MIC (efficacy) of these highly substituted eudesmane derivatives to assess their feasibility as drugs with a safe Therapeutic Index. This structural library confirms that while the core lactone warhead drives direct non-specific alkylation, the chemical functionalization of the eudesmane skeleton dictates the ultimate translational possibilities, providing a clear path to manipulate the balance between efficacy and toxicity.

#### 3.2.2 Sesquiterpenoid and monoterpenoid diversity non-SL synergistic metabolites

While the cinnamoyloxy eudesmane scaffold is the recognized chemotaxonomic marker, the *Verbesina* genus is, at its core, a dedicated biosynthetic factory for C_15_ molecules, exhibiting remarkable structural diversity that extends across multiple sesquiterpenoid skeletal types (Arciniegas et al., 2020). This complexity ensures that the core bioactive mechanism, the α-methylene-γ-lactone pharmacophore is deployed via numerous molecular architectures (Kaur et al., 2021; Ortega et al., 1985; Selener et al., 2019). Spreading the reactive lactone pharmacophore across diverse skeletal backbones alters non-covalent target engagement prior to covalent alkylation, directly influencing both efficacy and cell-line specificity. The biosynthesis of these diverse SLs often begins with the germacrane skeleton, which is widely considered the common precursor to the complex array of eudesmane derivatives (Bohlmann et al., 1980; akupovic et al., 1987). Evidence for this pathway includes the co-occurrence of germacrane derivatives alongside eudesmanes across many species (Bohlmann et al., 1980; 1982). A novel, active SL derivative, 6β-cinnamoyloxy-1β-hydroxy-10α-metoxy-3-oxo-germacra-4,5*Z*-ene, was structurally characterized following bioassay-guided isolation from the chloroformic extract of *V. turbacensis* (Mora et al., 2013).

This structural variability is not limited to bicyclic and macrocyclic scaffolds. Investigations into *Verbesina occidentalis Walter [Asteraceae]* revealed the presence of six new sesquiterpenes structurally elucidated as derivatives of muurolene, alongside Verboccidentafuran, a novel trisubstituted furan derivative (C_15_H_20_O) (Bohlmann and Lonitz, 1978). Furthermore, the genus’s reliance on multiple SL scaffolds includes the Elemanolides, such as Zempoalines C and D, isolated from *Verbesina seatonii* S.F.Blake [Asteraceae], whose highly characteristic elemane skeleton with a fused α,β-unsaturated-γ-lactone moiety was structurally established via X-ray crystallography (Ortega et al., 1985). Most recently, a comprehensive study on *V. sphaerocephala* successfully isolated six cadinene-type SLs, five of which were previously undescribed. The accurate structures of these compounds were elucidated by X-ray analyses, highlighting that the genus biosynthesizes multiple SL scaffolds from common C_15_ precursors, increasing the probability of finding a lead molecule with a distinct and selective mechanism of action (Arciniegas et al., 2020; Salmon et al., 1985). The genus also biosynthesizes the unique amorphane sesquiterpene γ-lactol, Verocephol, from *V. sphaerocephala*, marking the first reported occurrence of a γ-lactol with an amorphane skeleton (Salmon et al., 1985).

Beyond the non-volatile SLs, the Essential Oil (EO) represents a volatile, lipophilic line of chemical defense (Heyerdahl-Viau et al., 2023) and is overwhelmingly dominated by sesquiterpenes (99.2% total) (Albuquerque et al., 2006; Bezerra et al., 2018). The EO of *V. macrophylla*, for example, is heavily dominated by the characteristic C_15_ sesquiterpenes Germacrene D (37.3%) and its derivative, Germacrene D-4-ol (17.0%) (Bezerra et al., 2018; Heyerdahl-Viau et al., 2023). Similarly, the EO of *Verbesina negrensis* Steyerm. [Asteraceae] is dominated by α-pinene (43.1%), a known antibacterial monoterpene. This structural partitioning of anti-infective activity into volatile like α-pinene and non-volatile SLs fractions is strategic: the mechanism of action for such monoterpenes typically involves the destruction of the cellular integrity of the microorganism, inhibiting respiration and ion transport, which offers an alternative pharmacological pathway to the Michael addition mechanism characteristic of SLs (Domínguez-Barradas et al., 2015). This dual pathway approach reduces absolute reliance on highly toxic alkylation cascades, providing a higher likelihood of therapeutic value in the extract’s natural state where volatile fractions can exhibit a wider, safer toxicological window. The consistent presence of these sesquiterpenes reinforces the centrality of the terpene pathway to the plant’s chemical strategy (Bezerra et al., 2018).

This diverse arsenal is synthesized and housed within specialized plant structures. Anatomical analysis of *V. macrophylla* revealed that lipids, terpenes, and alkaloids are physically manufactured and stored in specialized leaf secretory ducts via an elaborate, energy-intensive mechanism known as granulocrine secretion. This elaborate and conserved production mechanism underscores the fundamental, evolved importance of these secondary metabolites for the plant’s defense system (Bezerra et al., 2018) The complexity extends to C_10_ monoterpenoids, evidenced by the isolation of the C_10_ alcohol isoepicampherenol and bornyl esters like (−)-bornyl ferulate and (−)-bornyl *p*-coumarate from *V. rupestris* (Box and Chan, 1975). These bornyl hydroxycinnamic esters were also confirmed in *V. turbacensis* (Caballero-Gallardo et al., 2023; Ogungbe et al., 2010) demonstrating that the plant utilizes multiple chemical classes to exert its powerful pharmacological effects. From a translational perspective, these non-SL co-metabolites, phenolics, and bornyl conjugates can function as essential synergists or metabolic buffers; antioxidant buffering from accompanying phenolics may actively reduce or modulate the extreme oxidative stress caused by pure SL accumulation in healthy host tissue. However, this inherent diversity leads to high chemotypic variability across different populations, with the relative percentages of key sesquiterpenes fluctuating significantly depending on the plant’s maturity. This variability complicates standardization efforts, emphasizing the need for robust protocols to ensure the consistency and reproducibility of IC_50_ and MIC data (Albuquerque et al., 2006). Consequently, achieving reproducible Selectivity Indices demands strict analytical control to manage this chemotypic drift and isolate high-priority leads from fluctuating raw botanical backgrounds.

#### 3.2.3 Non-SL chemical arsenal and biosynthetic complexity

The total pharmacological profile of *Verbesina* extracts is a complex outcome of a dense and multi-layered chemical defense system that extends far beyond the dominant SLs (Fawzy et al., 2013; Kaur et al., 2022). Qualitative phytochemical screening confirms the simultaneous presence of nearly all major secondary metabolite classes including Alkaloids, Glycosides, Saponins, Phenols, Tannins, Flavonoids, and Terpenoids, a comprehensive chemical diversity that necessitates a thorough investigation of synergistic effects for accurate biological interpretation (Fawzy et al., 2013). Managing this multi-layered network represents a major translational milestone, as the chemical co-occurrence of selective antimicrobial scaffolds alongside non-selective cellular toxins dictates the baseline safety constraints of the raw extracts.

A critical challenge for advancing *Verbesina* metabolites to clinical application lies in managing the presence of guanidine alkaloids, a class chemically distinct from the dominant SLs (Botta et al., 2003a). This class presents a unique therapeutic duality. On one hand, toxicological investigations into *V. encelioides* confirmed the presence of the highly potent, non-selective toxin galegine (3-methyl-2-butenyl-guanidine) (Eichholzer et al., 1982; Oelrichs et al., 1981) which is responsible for severe sheep and cattle losses and is a documented hazard for grazing livestock (Mehal et al., 2023; Oelrichs et al., 1981). This toxin’s acute lethality underscores the non-selective nature of the raw plant’s defense system (Mehal et al., 2023). On the other hand, the genus yields therapeutically valuable analogs, notably from *Verbesina caracasana* B.L.Rob. and Greenm. [Asteraceae], including the monomeric hypotensive agent caracasanamide and the dimeric, cyclobutane-containing caracasandiamide (Carmignani et al., 1999; Corelli et al., 1996; Delle Monache et al., 1991; 1992; 1999; Monache et al., 1993). These specialized guanidino-amide alkaloids represent a significant chemical class for cardiovascular therapy (Botta et al., 2003a). The natural co-occurrence of the lethal toxin galegine with chemically related, but non-toxic supportive molecules like *N*-2,3-dihydroxy-3-methylbutyl-acetamide in *V. encelioides* provides strong proof-of-concept that selectively isolating a non-cytotoxic therapeutic agent is chemically feasible, emphasizing the imperative for rigorous bioassay-guided fractionation (Compagnone et al., 2008; Eichholzer et al., 1982).

The extract’s capacity for anti-inflammatory and antioxidant activity is substantially defined by a high content of phenolic compounds and flavonoids (Hernández-Pérez et al., 2025; Rodríguez-Valdovinos et al., 2021). The overall bioactivity is reinforced by the presence of a diverse class of flavonol diglycosides, including quercetin 3-galactoside-7-glucoside and quercetin 3-xyloside-7-glucoside from *V. encelioides* (Glennie and Jain, 1980; Gorja and Bandla, 2024) and the novel Rhamnocitrin-3-*O*-glucuronide from *Verbesina myriocephala* Sch. Bip. ex Klatt [Asteraceae] (Wagner et al., 1974). More recent analyses have identified high concentrations of the anti-inflammatory and antioxidant agent Rutin (quercetin-3-*O*-rhamnoglucoside) in *V. sphaerocephala* (de Veras et al., 2021) and catechin derivatives (catechin-3-glycoside isomers) and phylloflavan in *V. crocata* (Van Nuland et al., 2013). The biological role of these non-SL components is critical, as they include sitosterol derivatives which exhibit anti-inflammatory, antineoplastic, and immune-modulatory activities. This dense profile of phenolic acids such as, *p*-coumaric acid, gallic acid, caffeic acid, etc. (Mehal et al., 2023; Rodríguez-Valdovinos et al., 2021) is directly linked to the allelopathic activity of the genus; HPLC analysis of *V. encelioides* root leachate confirmed that phenolic compounds are functionally involved in the chemical interference with competing flora (Inderjit et al., 2000). Furthermore, early phytochemical work confirmed the presence of highly reactive phenolic conjugates, including caffeic acid ethyl ester (a compound previously unknown as a natural product) and caffeic acid borneol ester in the aerial parts of species like *Verbesina alternifolia* (L.) Britton ex Kearney [Asteraceae] and *Verbesina fastigiata* B.L.Rob. and Greenm. [Asteraceae] (syn. *Verbesina greenmanii* Urb.) (Bohlmann and Zdero, 1976).

The extensive chemical profile is confirmed by comparative spectroscopic analyses, such as Raman spectroscopy on wild species like *V. virginica*, which exhibits a distinctly measurable spectrum based on marker bands assigned to scaffold molecules such as phenylpropanoids and carotenoids, supporting the fundamental premise that these specialized secondary metabolites produce unique chemical fingerprints that correlate with the diverse biological activities reported (Farber et al., 2020).

The genus exhibits further remarkable biosynthetic capacity through the production of Triterpenoid Saponins (Verma et al., 2019). A comprehensive investigation of *V. virginica* yielded six new compounds (Verbesinosides derivatives) possessing a novel 15,27-cyclooleanane-type triterpenoid skeleton (Xu et al., 2009) distinct from the oleanolic acid derivative Copteroside E isolated from *Verbesina suncho* S.F.Blake [Asteraceae] (Cerda-García-Rojas et al., 2000). The presence of these saponins, along with other non-SL triterpenoids such as friedelin, epifriedelin, α- and β-amyrin, and lupeol in *V. encelioides* (Jain et al., 2009; Kaur et al., 2022; Sultana et al., 2018) is vital as they contribute to the extract’s complexity, with saponins potentially influencing membrane permeability and thereby enhancing the cellular penetration of other lipophilic compounds (Cerda-García-Rojas et al., 2000). This interactive architecture highlights that polar phenolics and membrane-altering triterpenoids act as antioxidant buffers or absorption enhancers, modulating the toxic oxidative stress spikes of pure SL fractions and altering host cell tolerance CC_50_.

This complex defensive system also includes a diverse range of Acetylenic compounds and their cyclized sulfur derivatives, the thiophenes, which are recognized as chemotaxonomic markers of the Heliantheae tribe (Christensen and Lam, 1991). The investigation of Mexican *Verbesina* species confirms the genus’s rich, yet structurally diverse, chemical composition, reinforcing the structural complexity of these specialized compounds. The roots of *V. angustifolia* contained the linear C_13_ polyacetylene pentainene, along with the diterpene acids. Similarly, the roots of *Verbesina oncophora* B.L.Rob. and Seaton [Asteraceae] yielded polyacetylene pentainene and a diterpene alcohol (Bohlmann and Zdero, 1976). A reinvestigation of the roots of *V. virginica* (collected in Louisiana) further yielded the ubiquitous polyacetylene pentainene and caryophyllene along with eudesmane derivatives (Bohlmann and Zdero, 1979). Thiophenes, such as C_13_-Monothiophenes and C_13_-Dithio compounds, are known for their strong biological activity due to their ROS-generating photoactivation potential (Christensen and Lam, 1991). The complete profile even extends to specialized heterocycles, evidenced by the isolation of the only known 2-isopropyliden-2*H*-benzofuran-3-one from *V. luetzelburgii* (Pergomet et al., 2017).

Recent analytical studies have significantly broadened the understanding of the genus’s therapeutic potential by identifying non-traditional bioactive classes. The neuroprotective activity of the *V. encelioides* flower extract, for example, is chemically linked to carotenoids, such as Spirilloxanthin (12.38%–13.24% of the total composition), and the diterpene lactone Ginkgolide A. These compounds demonstrate efficacy by binding to the enzyme Acetylcholinesterase (AChE) (Verma et al., 2019). Furthermore, the genus’s defensive strategy employs structural macromolecules, with biochemical analysis of *V. encelioides* identifying a low molecular weight 14 kDa protein characterized as a host defense compound exhibiting moderate antimicrobial activity (Divya Ramakrishnan et al., 2017). The roots of *V. encelioides* also contain fundamental biomolecules that contribute to the extract’s complexity, including the amino acids nor-leucine and ornithine and carbohydrates D-ribose and sucrose (Kaur et al., 2022; Sindhu et al., 2010).

The plant’s rich organic matter and protein profile is fundamentally linked to its bioactivity as a growth-promoting component (Velasco-Ramírez et al., 2022a). More fundamentally, the high performance is linked to the genus’ known content of organic matter, carbohydrates, and proteins, suggesting they provide essential growth-promoting components which complement the high-potency, often toxic, defensive nature of the Sesquiterpene Lactones. The bioactivity of the extracts is attributed to their complex profile of organic compounds and proteins, enabling enzymatic activation for nutrient assimilation. Although key minerals like Nitrate and Potassium were not detected in the aqueous extracts, the presence of other nutrients like Ammonia, Ca, Mg, and Phosphate was confirmed (Velasco-Ramírez et al., 2022b). This comprehensive chemical complexity is now leveraged for nanotechnology; the hot water extract of *V. encelioides* and the aqueous extract of *V. crocata* are successfully used for the “green synthesis” of Silver Nanoparticles (AgNPs), where the compounds facilitate the reduction and chelation/stabilization of the resulting nanoparticles through redox processes (Kushwaha and Malik, 2012; Ramírez-Herrera et al., 2025).

A critical discrepancy exists between the reported safety of *Verbesina* in traditional medicine and the high cytotoxic potential observed in laboratory isolates. This ‘safety paradox’ is largely a function of extraction polarity. Traditional preparations, predominantly aqueous decoctions or infusions, prioritize the extraction of polar, supportive metabolites such as phenolic acids and glycosides, while largely excluding the highly lipophilic, and often more toxic, sesquiterpene lactones (SLs) sequestered in glandular trichomes. Consequently, while ethnobotanical use validates the genus’s broad therapeutic potential, it also masks the inherent risks of the raw chemical arsenal. This necessitates a transition from whole-extract pharmacology to bioassay-guided isolation, focusing on the Selectivity Index (SI) to rigorously separate potent antimicrobial efficacy from the underlying risk of non-selective alkylation.

## 4 Cytotoxic activity of *verbesina* metabolites: focus on IC_50_ profiles

The inherent chemical reactivity of SLs, driven by the α-methylene-γ-lactone moiety, ensures their broad-spectrum biological activity, encompassing both antimicrobial and antiproliferative effects. While this potency is desirable, the historical lack of quantitative cytotoxic investigation has often relegated the genus to a preliminary research stage (Mora et al., 2013). Early studies noted that out of over thirty-eight phytochemically characterized *Verbesina* species, only four had been biologically investigated, with limited IC_50_ data available (Mora et al., 2013). Consequently, the primary objective of this section is to compile and analyze the recently available quantitative cytotoxicity data, providing a contemporary and rigorous assessment of the genus’s true antiproliferative potential against cancerous and non-cancerous models, thereby establishing the crucial host-toxicity component for the final Selectivity Index (SI) calculation.

### 4.1 Anticancer potential across cell lines

The cytotoxic potential of *Verbesina* compounds is confirmed by consistent activity across both crude extracts and purified isolates, validating the eudesmane scaffold as an inherently bioactive template. Methanolic extracts of *V. encelioides* demonstrated significant and quantifiable cytotoxic activity against diverse human cancer models, displaying highly consistent potency across three tumor cell lines after 48 h of exposure. The extract achieved half-maximal growth inhibition (GI_50_) values of 26.32 ± 2.42 μg/L against MCF-7 (Breast Adenocarcinoma), 28.65 ± 2.89 μg/L against NCI-H460 (Non-Small Lung Cancer), and 26.82 ± 8.54 μg/L against SF-268 (CNS Cancer). This consistency across disparate cancer origins suggests a broad-acting, fundamental cytotoxic mechanism, which is a hallmark of the reactive SLs that define the genus’s chemical profile (Albalawi et al., 2015).

However, efficacy is strongly dependent on the extraction method used. Comparative studies using both ethanolic (VE) and aqueous (VA) crude extracts of *V. encelioides* against the human colon cancer cell line HCT-116 revealed a stark difference in potency. The ethanolic extract (VE) exhibited significantly higher potency with an IC_50_ of 310 μg/mL (0.31 mg/mL), which was twice as potent as the aqueous extract (VA) at 650 μg/mL (0.65 mg/mL). This difference confirms the strategic necessity of concentrating the lipophilic compounds, primarily Sesquiterpene Lactones and triterpenoids, through non-polar extraction methods to maximize the yield of active lead molecules (Farshori, 2021).

The intrinsic activity of the core eudesmane scaffold itself, even before cinnamoyl esterification, is confirmed by low micromolar cytotoxicity. The key sesquiterpenediol, Verbesindiol (4α, 6β-dihydroxyeudesmane), isolated from *V. virginica*, demonstrated potent activity against advanced, castration-resistant prostate cancer cell lines. Specifically, Verbesindiol achieved an IC_50_ of 4.0 ± 0.6 μM against PC-3 (Androgen-independent) and 5.2 ± 0.6 μM against DU 145 (Androgen-independent) cells. This demonstrates that the parent eudesmane skeleton is inherently cytotoxic, positioning Verbesinol derivatives as excellent starting points for SAR investigation aimed at optimizing selectivity (Shaffer et al., 2016).

Crucially, isolated SLs, the primary focus of this review, also demonstrate high potency. The core eudesmane scaffold, 4β-cinnamoyloxy,1β,3α-dihydroxyeudesm-7,8-ene, exhibited potent antiproliferative activity (GI_50_) against a broad panel of human tumor cell lines, with values ranging from 14.7 μM to 32.3 μM (Dalla Via et al., 2015). This finding is complemented by related research on *V. persicifolia*, which isolated an eudesmane triol mono-cinnamate reported to display promising anti-inflammatory, anti-hypoglycemic, and powerful peroxynitrite scavenging activities, along with measurable cytotoxicity against different tumor cell lines. This array of activity across multiple compounds in *V. persicifolia* directly establishes it as a major source of selectively toxic scaffolds (Cadanedo et al., 2015). This translational potential is further substantiated by a Mexican patent detailing the isolation of the eudesmane 4CDE (a core scaffold), which is claimed for the treatment of diabetes mellitus type 2, cancer, obesity, and as an anti-inflammatory, directly validating the high-value, multifaceted application of this genus (Jimenez Estrada and Vazquez Candanedo, 2015).

However, not all crude extracts exhibit high cytotoxicity. The hydroethanolic extract from the stem of *V. turbacensis* (a species traditionally used for inflammation) demonstrated a low cytotoxic effect against the human hepatoma cell line HepG2. After 24 h of exposure, the extract showed minimal toxicity (IC_50_ > 500 μg/mL), and even after 48 h, the IC_50_ remained relatively low at 334.6 μg/mL. This low-to-moderate cytotoxicity reinforces the argument that the high-potency effects of SLs are often diluted in crude hydroethanolic extracts, necessitating chemical purification to isolate the potent molecules (Caballero-Gallardo et al., 2023).

Finally, the potential for cellular specificity in the crude extracts is an important consideration for SI optimization. The alcoholic extract of *V. encelioides* exhibited a differential cytotoxic response across human cancer cell lines. While the extract was strongly cytotoxic to HepG2 cells, reducing viability to 53% at 1,000 μg/mL, it proved non-cytotoxic to A-549 (human lung cancer) cells, even at the highest concentrations tested. This inherent degree of cellular specificity suggests that isolating key compounds may yield a selective drug candidate with a favorable SI (Al-Oqail et al., 2016).

### 4.2 Molecular mechanisms of cytotoxicity

**FIGURE 3:**
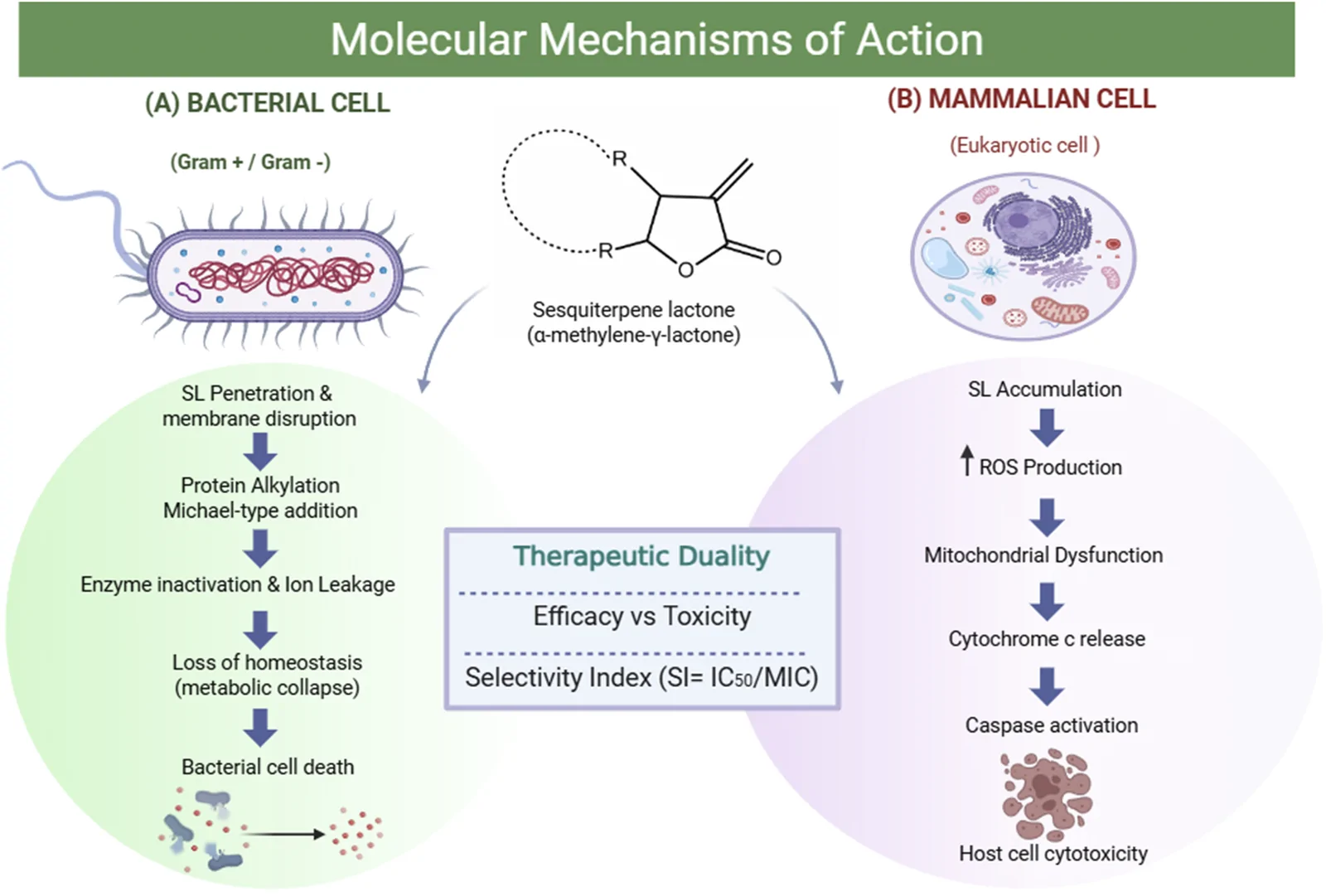
Dual molecular mechanisms of action of Verbesina sesquiterpene lactones. The α-methylene-γ-lactone pharmacophore mediates distinct biological effects depending on the cellular target. **(A)** Bacterial cell: sesquiterpene lactones penetrate and disrupt the bacterial membrane, alkylate thiol-containing proteins via Michael-type addition, cause enzyme inactivation and ion leakage, and ultimately lead to loss of metabolic homeostasis and bacterial cell death. **(B)** Mammalian cell: intracellular accumulation of sesquiterpene lactones promotes reactive oxygen species (ROS) overproduction, mitochondrial dysfunction, cytochrome c release, caspase activation, and apoptosis, resulting in host cell cytotoxicity. This mechanistic asymmetry underlies the therapeutic duality of these compounds and highlights the importance of the selectivity index (SI = IC_50_/MIC) for translational feasibility.

The potent IC_50_ profiles observed in *Verbesina* metabolites are fundamentally linked to the inherent, non-selective chemical reactivity of the SLs, which is primarily executed through mitochondria-mediated apoptosis induced by the generation of Reactive Oxygen Species (ROS) (Al-Oqail et al., 2016; Farshori, 2021). This mechanism is consistent with the predicted alkylating action of the α-methylene-γ-lactone pharmacophore against nucleophilic sites on cellular proteins and peptides (Figure 3) (Farshori, 2021). From a standpoint of translational feasibility, mapping these cytotoxic mechanisms is not merely an exercise in characterizing cell death, but a necessary prerequisite to decode the ‘chemical duality’ of the genus. By defining the exact molecular parameters that drive mammalian host toxicity (IC_50_), these mechanisms reveal the structural limits within which the *α*-methylene-*γ*-lactone core must be optimized to achieve a competitively high Selectivity Index (SI = IC_50_/MIC).

#### 4.2.1 Oxidative stress and the apoptotic cascade

Mechanistic studies using the ethanolic extract (VE) of *V. encelioides* against the HCT-116 colon cancer cell line definitively established the full cascade of cell death. The initial event involves oxidative stress induction; exposure to the VE significantly increased the level of Lipid Peroxidation (LPO) (up to 74%) while simultaneously causing a substantial Glutathione (GSH) depletion (up to 46%), strongly indicating a pro-oxidant mechanism. This chemical stress directly leads to a dose-dependent surge in intracellular ROS production, which subsequently disrupts the integrity of the mitochondrial membrane. This disruption results in a significant loss of Mitochondrial Membrane Potential (MMP) (up to 54% decline), leading to mitochondrial damage (Farshori, 2021).

The cytotoxic mechanism is further confirmed at the molecular level. The disruption of the MMP triggers a classic apoptotic cascade, characterized by the significant upregulation of pro-apoptotic genes. Specifically, the extract significantly upregulated the expression of Bax (by 2.1-fold), p53 (by 2.9-fold), caspase-3 (by 2.2-fold), and caspase-9 (by 2.5-fold), while simultaneously downregulating the anti-apoptotic gene Bcl-2 (by 0.55-fold). The resulting activation of caspases confirms that programmed cell death is the primary mode of action (Farshori, 2021). This mechanism is consistent with separate studies on the alcoholic extract of *V. encelioides* against HepG2 cells, which also found that the primary cause of cell death was oxidative stress, resulting in mitochondrial damage and genotoxic potential evidenced by extensive DNA damage quantified by the Comet Assay. This DNA damage likely triggers the observed G_2_/M cell cycle arrest (up to ∼50% at 1,000 μg/mL), leading to cell death upon repair failure (Al-Oqail et al., 2016). Importantly, this mechanism appears to be non-folate related, as the *V. encelioides* methanol extract displayed no inhibitory effect on DHFR enzyme activity, supporting the hypothesis that the cytotoxic action proceeds via non-enzymatic Michael addition rather than competitive enzyme antagonism. This non-enzymatic Michael addition underpins the challenge of non-selective toxicity; because alkylation targets conserved nucleophilic residues common to both microbial and mammalian targets, raw extracts inevitably present narrow, unoptimized SI margins that restrict their direct translational possibilities unless structural or formulation-driven optimization is applied.

#### 4.2.2 Scaffold dependency and mitochondrial targeting

Mechanistic studies focusing on isolated pure eudesmane scaffolds confirm that the cytotoxic mechanism is definitively mapped to the mitochondria, but the mode of action is structure-specific, a crucial finding for SAR and SI optimization. One identified mechanism involves mild uncoupling of the respiratory chain. Studies on the pure eudesmane CDE (4β-cinnamoyloxy,1β, 3α-dihydroxyeudesm-7,8-ene), a characteristic scaffold of the genus, revealed that it acts as a natural mild uncoupler in rat liver mitochondria (RLM). This effect involves CDE inducing a drop in the mitochondrial membrane potential (ΔΨ) and causing a bioenergetic collapse. Mechanistically, CDE is proposed to interact with the respiratory chain, particularly at the level of cytochrome C oxidase (COX), leading to the generation of H_2_O_2_ and subsequent ROS production. This critical finding confirms that the cytotoxic or anti-proliferative action of *Verbesina* SLs is not confined to simple alkylation but includes complex regulation of cellular energy metabolism, a crucial pathway in both cancer and microbial persistence (Dalla Via et al., 2014).

**FIGURE 4:**
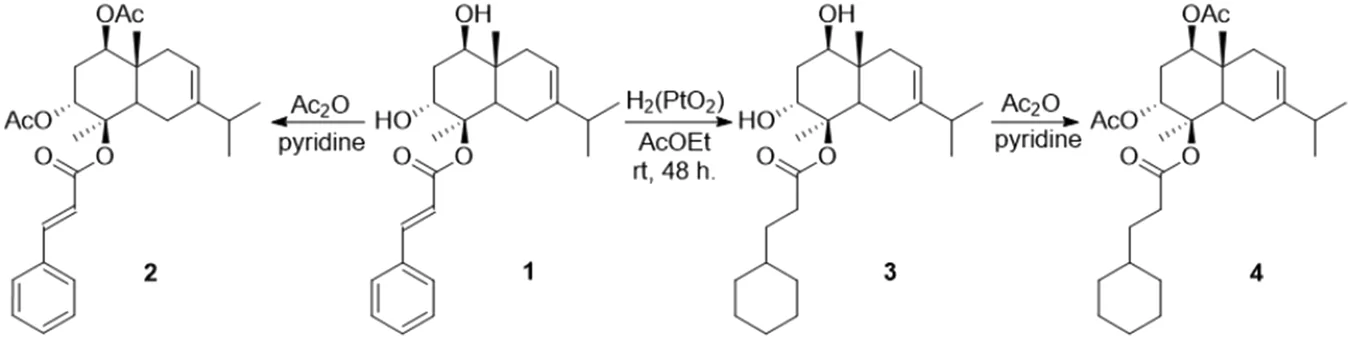
Synthesis of 4β-cinnamoyloxy,1β,3α-dihydroxyeudesm-7,8-ene derivatives **2**–**4**.

A second, more nuanced mechanism was revealed by the comprehensive mechanistic investigation of 4β-cinnamoyloxy,1β,3α-dihydroxyeudesm-7,8-ene and its derivatives (Compound **1**), which differentiated the mode of action based on subtle functionalization of the eudesmane skeleton. This work identified two distinct pathways for apoptosis. The first pathway involves Mitochondrial Permeability Transition (MPT) induction: the native hydroxyl groups on the eudesmane moiety (found in the lead Compound **1** and the hydrogenated Compound **3**) were found to be crucial for inducing Ca^2+^-dependent MPT, which leads to mitochondrial swelling and the release of pro-apoptotic factors (Cyt C and AIF). The second pathway involves Respiratory Chain Inhibition: conversely, when the hydroxyl groups were masked by acetate substituents (in the diacetate Compound **2** and hydrogenated diacetate Compound **4**), the mechanism shifted dramatically. These acetylated compounds caused injury by directly inhibiting Succinate dehydrogenase of the mitochondrial respiratory chain and inducing a general uncoupling effect on oxidative phosphorylation (Figure 4). This complex mechanistic picture demonstrates that subtle structural modifications on the eudesmane skeleton drastically alter the cellular target, without necessarily abolishing baseline bioactivity. Masking or exposing specific functional groups provides medicinal chemists with a reliable dial to strategically optimize the SI, decoupling general mammalian cellular collapse from targeted microbial disruption to enhance overall translational feasibility. (Dalla Via et al., 2015).

### 4.3 Long-term cytotoxic profile

Understanding the long-term cytotoxic profile of these scaffolds is essential for translating acute IC_50_ data into a true therapeutic advantage. Further mechanistic studies on Verbesindiol, the non-esterified core scaffold, revealed a complex profile. While the compound demonstrated high acute cytotoxicity (84%–97% cell death) in the SRB assay, suggesting excellent initial anti-cancer potency, clonogenic assays (which measure the ability of cancer cells to form colonies after drug washout) showed that compound 6 had no detectable effects on colony-forming ability. This surprising result suggests that the initial cytotoxic mechanism of Verbesindiol does not prevent the long-term regenerative capacity of the cells, differentiating its mode of action from other *Verbesina* metabolites that do cause G_2_/M arrest and inhibit colony formation. From an anti-resistance perspective, this structural behavior represents a critical translational roadblock: if acute exposure triggers high host cell toxicity (IC_50_) but fails to cause persistent long-term growth arrest or cell eradication, the scaffold risks triggering narrow clinical windows. This complexity emphasizes that achieving a true therapeutic advantage relies not just on acute transient IC_50_/MIC ratios, but on verifying that the underlaying structural patterns favor permanent target favor permanent target eradication over reversible metabolic injury (Shaffer et al., 2016).

### 4.4 Toxicity to non-cancerous cells

The determination of a favorable SI is the central translational challenge for *Verbesina* metabolites, a necessity rooted in the genus’s long-standing documentation as a poisonous plant (Oelrichs et al., 1981; Sharma et al., 2018). This history provides the historical imperative for rigorous toxicity screening (IC_50_/CC_50_) in modern pharmacology (Oelrichs et al., 1981). The non-selective and potentially fatal nature of the crude plant material is demonstrated by the potent guanidine alkaloid, galegine (3-methyl-2-butenyl-guanidine), which is classified as a documented hazard for grazing livestock in the United States. Exposure to galegine causes severe pathological effects and death in sheep, with observed symptoms consistent with congestion of the lungs and fluid in the chest cavity (Kaur et al., 2022; Oelrichs et al., 1981). The high cytotoxic activity of this known poison is confirmed by laboratory testing, where isolated Galegine was found to be highly potent against the HepG2 (Hepatocellular Carcinoma) cell line, exhibiting an IC_50_ value of 10.9 μg/ML (Abbas et al., 2016). This high degree of cytotoxicity for a known poison further underscores the absolute necessity of rigorous bioassay-guided isolation and chemical optimization to establish sufficient SI for any therapeutic lead compound (Mehal et al., 2023). The historical toxicological findings also revealed a crucial co-occurrence of these non-selective toxins with chemically related, but experimentally verified non-toxic supportive molecules, such as the *N*-2,3-dihydroxy-3-methylbutyl-acetamide isolated alongside galegine in *V. encelioides*. This functional differentiation provides crucial proof-of-concept that selectively isolating a non-cytotoxic therapeutic agent from the complex crude mixture is chemically feasible (Eichholzer et al., 1982).

The core challenge remains the lack of specificity inherent to the reactive SLs. Quantitative data on the core eudesmane scaffold, Compound **1** (4β-cinnamoyloxy, 1β,3α-dihydroxyeudesm-7,8-ene) (Figure 4), demonstrated a pronounced lack of specificity toward tumor cell lines and the non-tumorigenic human mesothelial cells (MeT-5A). The GI_50_ value for Compound **1** on the non-tumorigenic MeT-5A cells (32.3 μM) was found to be directly within the range of its toxicity against tumor cells (14.7 μM against A431). This direct demonstration of non-selective cytotoxicity underscores the central challenge of the entire review: the necessity of maximizing the efficacy (MIC) while minimizing the host toxicity (IC_50_ to healthy cells) to achieve a favorable SI. The necessity for further toxicity evaluation remains a critical concern for the translational use of all *Verbesina* extracts; while some aqueous extracts of *V. crocata* demonstrated diuretic activity without obvious signs of toxicity in rats, the probable presence of the potent toxin galegine makes the overall toxicity of the plant a primary barrier to advancing novel scaffolds (Heyerdahl-Viau et al., 2023). Furthermore, the strong allelopathic history of the genus provides a clear historical and contemporary imperative for rigorous toxicity screening (Mehal et al., 2023).

Despite these concerns, quantitative data strongly supports the hypothesis that selective separation of the therapeutic scaffold can yield low-toxicity agents. An investigation into the antiprotozoal activity of *V. encelioides* isolated six major constituents, including the triterpenoids pseudotaraxasterol and its derivatives, and β-sitosterol glycosides. When tested against human embryonic lung fibroblasts (MRC-5), a standard non-cancerous cell line, all isolated compounds were determined to be non-cytotoxic with high values (CC_50_ > 64 μg/mL). This result contrasts sharply with the activity of many conventional chemotherapy agents and provides crucial evidence of the genus’s potential for a favorable SI. This finding is supported by the study of the crude alcoholic extract of *V. encelioides*, which showed a strong cytotoxic response against HepG2 and MCF-7 cancer cells but was non-cytotoxic to A-549 (human lung cancer) cells. This differential response to specific cell lines suggests an inherent degree of cellular specificity, indicating that isolating key compounds may yield a selective drug candidate (Al-Oqail et al., 2016).

Furthermore, the toxicity of the EO fraction, which is dominated by sesquiterpene hydrocarbons, also demonstrates a favorable safety profile. The acute toxicity of the *V. macrophylla* EO was evaluated in mice, with the median lethal dose (LD_50_) estimated to be greater than 5,000 mg/kg (p.o.). The oil exhibited a low rate of hemolysis in human red blood cells (hRBCs), with the highest rate being only 2.14% ± 0.10% at 5,000 μg/mL. A hemolysis rate of less than 5% is considered a favorable safety indicator for drug application, suggesting that the EO compounds, despite being lipophilic, interact minimally with the human cellular membrane (de Veras et al., 2021). The necessity of balancing efficacy with host safety is further reinforced by reports that anthelmintic activity has been linked to high levels of the toxic guanidine alkaloid, galegine, and nitrates in *Verbesina* alcoholic extracts, reinforcing the core challenge for developing a safe therapeutic agent: maximizing efficacy while rigorously ensuring the selective isolation of compounds away from known toxins like galegine (Abdel-Wahab et al., 2017).

## 5 Antimicrobial efficacy and anti-AMR mechanisms

The established chemical arsenal of the *Verbesina* genus, dominated by SLs, C_13_ polyacetylenes, and volatile terpenes, translates directly into a potent, broad-spectrum defense system that provides activity against bacterial, fungal, protozoal, and parasitic pathogens. This inherent chemical capability supports the ethnopharmacological use of *Verbesina* species in treating infections and wounds. The rigorous quantification of this efficacy, often expressed as the MIC, is the critical second metric required to calculate the SI and validate the translational potential of its specialized metabolites (Mora et al., 2013).

### 5.1 Activity against clinically relevant pathogens

To provide a critical hierarchy of the genus’s anti-infective potential, the reported metabolites can be ranked based on their minimum inhibitory concentrations (MIC). The eudesmane-type SLs (particularly cinnamoyloxy derivatives) represent the primary antimicrobial leads of the genus, achieving high-potency MIC_100_ values as low as 3.9–5.0 μg/mL. Secondary activity is attributed to the volatile sesquiterpene fraction (essential oils), which demonstrates broad-spectrum but moderate inhibition, with MICs typically ranging from 16 to 512 μg/mL. Finally, phenolic and flavonoid compounds are categorized as tertiary supportive metabolites; while their direct MICs are generally higher, they provide essential synergistic antioxidants and anti-inflammatory effects that enhance the overall host-mediated therapeutic profile. This broad-spectrum defense system is confirmed across numerous pathogenic kingdoms, ranging from human pathogens to agricultural pests. Even prior to chemical enhancement, crude extracts of *V. encelioides* demonstrated reliable activity, validating their initial use in drug discovery screens. For instance, crude methanolic extracts of *V. encelioides* exhibited effectiveness against the bacteria *Escherichia coli* and *Vibrio cholerae*, as well as the fungi *Aspergillus flavus* and *Aspergillus niger*. This robust baseline activity suggests that the chemical potency of the genus’s natural metabolites alone is sufficient to warrant further investigation and optimization (Bhati-Kushwaha and Malik, 2013).

Furthermore, the potent anti-fungal properties are highlighted in a process patent that claims the use of *V. encelioides* extract for treating dermatophyte infections. The extracts demonstrated anti-mycotic activity against clinically relevant fungi, including *Microsporum canis*, *Trichophyton Rubrum*, and *Malassezia furfur*, providing quantitative *in vitro* validation of the genus’s broad-spectrum anti-infective capacity (Wankhade and Rai, 2010). The total hydroalcoholic extract and its resulting fractions possess significant and varied antimicrobial properties, demonstrating high activity against Gram-positive bacteria (*Staphylococcus aureus* and *Bacillus subtilis*) and fungi (*Aspergillus fumigatus* and *Candida albicans*), while showing moderate activity against the Gram-negative bacterium *E. coli*. Notably, the total hydroalcoholic extract exhibited antifungal activity against *C. albicans* superior to the reference drug Amphotericin B in the diffusion agar technique. The ethyl acetate fraction generally maintained higher activity than the petroleum ether fraction in the antimicrobial assays, suggesting the concentration of moderately polar, active principles (Abbas et al., 2016).

The efficacy of isolated SLs, the focus of this review, is measured in the low micromolar range. The pure isolated germacrene SL exhibited measurable antibacterial activity against two clinically relevant Gram-positive pathogens, with the MIC determined to be 64 μg/mL against both *S. aureus* (ATCC 29213) and *Enterococcus faecalis* (ATCC 29212) (Mora et al., 2013). SLs isolated from *Verbesina* species also demonstrate significant activity against fungi and oomycetes, showcasing a broad-spectrum anti-infective capacity. In the study of *Verbesina lanata* B.L.Rob. and Greenm. [Asteraceae], the crude ethyl acetate extract showed a MIC_100_ of 35 μg/mL against *P. viticola* (grapevine downy mildew). Importantly, when tested as pure compounds, several isolated eudesmane SLs showed MIC_100_ values below that of the crude extract, confirming they are the primary active principles. The high potency of these pure SLs is critical, as exemplified by compounds such as 6β-cinnamoyloxy-3β,4α-dihydroxyeudesmane, which achieved an MIC_100_ of 3.9 μg/mL against *Plasmopara viticola*, and 6β-cinnamoyloxy-1β-hydroxyeudesm-4-en-3-one, with an MIC_100_ of 5.0 μg/mL. This potency, up to 7-fold greater than the crude extract, highlights the necessity of purification for maximizing efficacy and accurately calculating the Therapeutic Index. The eudesmane sesquiterpenes were further confirmed as the primary active constituents responsible for significant antifungal activity against *P. viticola*, with five compounds achieving MIC_100_ values less than 10 μg/mL (Ramseyer et al., 2017).

The EO fraction, which contains volatile terpenes, also demonstrates significant, quantifiable inhibitory effects. The EO from *V. negrensis* confirmed efficacy against common human pathogens, achieving an MIC of 500 μL/mL against *S. aureus* (ATCC 25923) and 350 μL/mL against *E. faecalis* (ATCC 29212). While this EO showed no activity against Gram-negative bacteria such as *E. coli* or *K. pneumoniae* at the tested concentrations, the confirmed efficacy against Gram-positive pathogens is consistent with broader *Asteraceae* literature (Mora et al., 2015). The essential oil of *V. macrophylla* showed both antibacterial and antifungal activity against strains causing respiratory and urinary tract infections, with MIC values varying from 8 to 512 μg/mL for bacteria and 4 to 32 μg/mL for *C. albicans*. Notably, the EO’s activity against *Klebsiella pneumoniae* (MIC 16 μg/mL) was observed to be superior to the standard antibiotic Amikacin (MIC 32 μg/mL). Furthermore, it achieved potent antifungal activity against *C. albicans* (MIC 4 μg/mL) (de Veras et al., 2021).

Beyond bacteria, *Verbesina* compounds demonstrate broad-spectrum activity against protozoal and parasitic agents (Ezzat et al., 2017). Studies on the Argentinean species *V. subcordata* showed significant trypanocidal activity against *T. cruzi* (the causative agent of Chagas’ disease), with the non-polar dichloromethane extract achieving 97.7% ± 0.2% growth inhibition at 100 μg/mL, suggesting that the lipophilic SLs and terpenoids are responsible for the parasiticidal effect (Selener et al., 2019). Expanding on this, aqueous extracts of the native Brazilian species *V. macrophylla* exhibit potent activity against the same protozoan, eliminating 100% of the epimastigote form of *Trypanosoma cruzi* after treatment with a moderate concentration of 200 μg/mL. The inflorescence extract also demonstrated significant fungicidal efficacy, inhibiting the growth of *C. tropicalis* by 41% (Bezerra et al., 2024). Activity against other Neglected Tropical Diseases (NTDs) is also moderate; for instance, benzyl 2,6-dimethoxy benzoate from *V. encelioides* showed an IC_50_ of 38.1 μg/mL against *Trypanosoma brucei* (Ezzat et al., 2017). The genus also provides strong efficacy in agricultural biocontrol, demonstrating strong nematicidal activity against the economically destructive root-knot nematode, *Meloidogyne javanica*. The most potent activity was found in the aqueous extract of fresh flowers of *V. encelioides*, and incorporating the plant material into nematode-infested soil significantly reduced the number of viable juvenile nematodes (Kaur et al., 2022; Oka, 2012).

Finally, non-small molecule defense components confirm that multiple fractions of *Verbesina* exhibit broad-spectrum activity. An isolated 14 kDa protein from *V. encelioides* validated the susceptibility of multiple pathogens through zone of inhibition assays, confirming activity across Gram-negative bacteria (*E. coli*, *Plasmopara aeruginosa*), Gram-positive bacteria (*S. aureus*, *E. faecalis*), and Fungal Pathogens (*A. flavus*, *F. oxysporum*) (Divya Ramakrishnan et al., 2017). The extracts also demonstrate potent antifungal activity against the problematic necrotrophic fungus *Botrytis cinerea*, with the aqueous stem extract exhibiting the highest antifungal activity (IC_50_ of 0.10 mg/mL) (Rodríguez-Valdovinos et al., 2024).

Furthermore, several Verbesina species demonstrate significant *in vitro* activity against both Gram-positive and Gram-negative bacteria, directly supporting their potential as anti-infective agents. Methanolic extracts of *V. sphaerocephala* exhibited remarkable antibacterial efficacy, demonstrating growth inhibition that was statistically non-significant from the ampicillin positive control (10 mg/mL) in several tests. Against *E. coli* (Gram-negative), the leaf extract achieved 95.00% ± 5.00% growth inhibition, and against *S. aureus* (Gram-positive), the leaf extract showed 90.00% ± 5.00% growth inhibition (Rodríguez-Valdovinos et al., 2021). Interestingly, the study suggested that the antimicrobial effect was negatively correlated with the total phenolic and flavonoid content, implying that less abundant, possibly more potent, lipophilic compounds, such as the characteristic Sesquiterpene Lactones may be the primary drivers of the antibacterial action, or that a synergistic effect among the diverse metabolites is present.

The efficacy of this wide-ranging arsenal is reinforced by the conclusion that the plant’s overall anti-infective potency relies heavily on a synergistic effect between its many constituents, including SLs, triterpenoids, and flavonoids (Ezzat et al., 2017). Furthermore, the widespread crisis of anthelmintic resistance necessitates alternative control strategies, for which *Verbesina* extracts provide a promising phytomedicine source. The anthelmintic activity in the Egyptian context has previously been linked to high levels of the toxic guanidine alkaloid, galegine, and nitrates in *Verbesina* alcoholic extracts. This reinforces the core challenge for developing a safe therapeutic agent: maximizing anti-parasitic efficacy while rigorously ensuring the selective isolation of compounds away from known toxins like galegine (Abdel-Wahab et al., 2017).

### 5.2 Molecular mechanisms of antimicrobial action

The anti-infective efficacy observed in *Verbesina* extracts is the result of a multi-layered defense strategy, employing distinct mechanisms to disrupt fundamental processes in diverse microbial kingdoms (Mora et al., 2013). While the cytotoxic mechanism of SLs is primarily through Michael addition to nucleophilic proteins, the genus deploys non-SL metabolites that operate through separate cytotoxic pathways, often resulting in complex synergistic interactions.

#### 5.2.1 The core mechanistic hypothesis: alkylation and membrane disruption

The primary hypothesis for SL action in microbial systems mirrors that in mammalian cytotoxicity: the highly reactive α-methylene-γ-lactone moiety acts as a non-specific alkylating agent. This mechanism, which involves non-enzymatic Michael addition to nucleophilic sites on biological proteins, distinguishes the SLs from many conventional antibiotics that target specific enzymes like DHFR (dihydrofolate reductase). Studies have demonstrated that the *V. encelioides* methanol extract displayed no inhibitory effect on DHFR enzyme activity despite robust cytotoxic efficacy, strongly supporting this non-enzymatic mechanism (Albalawi et al., 2015).

This alkylation mechanism leads to significant structural damage to the target cells. For instance, ultrastructural analysis of *T. cruzi* epimastigotes exposed to *V. macrophylla* extract revealed extensive damage, including cytoplasmic and nuclear disorganization, vacuolization, and swelling of reservosomes. This disruption, confirmed via Sytox Green fluorescence, indicates a compromised plasma membrane. The mode of action, disrupting the crucial cell membrane barrier reinforces the importance of identifying compounds that bypass conventional resistance pathways. Researchers strongly suggest that the sesquiterpene lactones present in *V. macrophylla* contribute to this membrane-disrupting and *T. cruzi* activity, further establishing the SL class as the driver of anti-infective effects in the genus (Bezerra et al., 2024).

#### 5.2.2 Specialized mechanisms of non-SL metabolites

The broad spectrum of activity observed in crude extracts (cytotoxicity, anthelmintic, and anti-fungal) relies heavily on the synergistic interaction of diverse metabolites, each acting via a different, specialized mechanism (Bohlmann and Zdero, 1976). This co-occurrence of various chemical classes suggests a multi-layered defense and multiple modes of action that contribute to the overall biological effects observed in crude extracts. For instance, the bornyl caffeic acid ester and related compounds combine a terpene scaffold with a phenolic acid, structures known for antioxidant and anti-inflammatory roles, hinting at complex synergistic interactions between the various metabolites (Bohlmann and Zdero, 1976). Other specialized metabolites rely on light-activated mechanisms for cytotoxicity. Thiophenes (sulfur-containing acetylenes) are highly susceptible to photoactivation, generating singlet oxygen and other ROS when exposed to UV-A light. This mechanism leads to broad-spectrum toxicity, often by causing irreparable damage to the microbial cell membrane and DNA (Christensen and Lam, 1991).

The anti-protozoal efficacy of Verbesina compounds is supported by targeted molecular mechanisms beyond non-selective cell damage. The bornyl esters isolated from *V. turbacensis* were found to possess potent inhibitory activity against the cysteine protease rhodesain. Rhodesain is the major cysteine protease utilized by the parasite Trypanosoma brucei rhodesiense (the causative agent of African trypanosomiasis/sleeping sickness). This targeted enzyme inhibition confirms that Verbesina metabolites can achieve high efficacy by directly interfering with a specific, critical virulence factor of a neglected tropical pathogen (Ogungbe et al., 2010).

Other specialized metabolites rely on light-activated mechanisms for cytotoxicity. Thiophenes (sulfur-containing acetylenes) are highly susceptible to photoactivation, generating singlet oxygen and other ROS when exposed to UV-A light. This mechanism leads to broad-spectrum toxicity, including anti-fungal and anti-bacterial activity, often by causing irreparable damage to the microbial cell membrane and DNA. The inclusion of these photoactive metabolites in Verbesina extracts further supports the hypothesis that the gross biological effect is a complex synergistic interaction between multiple compound classes, each operating via a distinct mechanism (Christensen and Lam, 1991).

The oil extract of *V. alternifolia*, which demonstrated potent adulticidal anthelmintic properties against the equine parasite *Habronema muscae*, revealed a mechanism linked to physical and nutritional interference. Scanning Electron Microscopy (SEM) analysis revealed irreversible degenerative changes to the parasite’s external morphology. Changes included shrinking, detachment, and distortion of the cuticle, the basic entry route for anthelmintic drugs and deformation of the lips and papillae. This mechanism suggests that the active components, attributed to the presence of terpenoids, flavonoids, and aromatic compounds, facilitate their effect through passive diffusion and subsequent membrane and structural disruption. Furthermore, the formation of aggregates in and around the buccal capsule (anterior digestive tract) suggests a disruptive action that may interfere with the nematode’s nutrition, potentially leading to undernourishment and mortality, similar to the proposed action of SLs targeting cellular processes (Abdel-Wahab et al., 2017).

The antifungal mechanism is also multi-faceted. The fungicidal effect against Botrytis cinerea is hypothesized to be due to a synergistic effect of the identified phenolic compounds. Individual phenolics present disrupt key fungal processes in multiple ways: Protocatechuic acid affects membrane permeability; Vanillic acid inhibits ergosterol synthesis; and Quercetin alters cell membrane composition through oxidative stress (Rodríguez-Valdovinos et al., 2024). Similarly, the potent anti-cyanobacterial effect of the aqueous extract of *V. encelioides* against Microcystis aeruginosa involves significant physiological disruption, causing a marked decrease in the content of Chlorophyll-a and carotenoids, demonstrating that the compounds interfere with photosynthesis and ultimately induce cell death (Lahlali et al., 2023).

#### 5.2.3 Ecological and biosynthetic context

The sheer chemical arsenal confirmed throughout the genus is a functional manifestation of potent allelopathic properties. The economic and ecological pressure exerted by *V. encelioides* is directly linked to the use of defensive secondary compounds that enable it to resist both pests and competing flora (Grichar and Sestak, 1998). This allelopathic effect suggests that the active metabolites, whether SLs or acetylenic compounds have mechanisms of action capable of disrupting fundamental cell survival processes (membrane integrity and energy metabolism) that are common across diverse microbial kingdoms (Al-Sodany et al., 2018; Fawzy et al., 2013).

The defense mechanism is further supported by direct anatomical evidence: Histochemical staining confirms that the synthesis and storage of Verbesina’s key metabolites occur directly within specialized glandular trichomes. These trichomes actively accumulate terpenoids (including sesquiterpenes) and phenolic/fluorescent metabolites. This direct evidence of a structural defense system confirms that the compounds responsible for antimicrobial activity and cytotoxicity are natural defensive agents designed for broad-spectrum biotic challenge (Aschenbrenner et al., 2013). The biosynthetic process supporting this defense is highly elaborate. In *V. macrophylla*, specialized leaf secretory ducts actively produce and store defensive materials, including terpenes and alkaloids, to combat herbivores and microbes via a complex process known as granulocrine secretion. This elaborate, energy-intensive mechanism, where secretion begins in the plastids of specialized transfer cells, confirms that anti-infective and anti-herbivory activities are a primary ecological function of the *Verbesina* genus, supporting the hypothesis that these compounds possess powerful, broad-spectrum bioactivity (Bezerra et al., 2018). This non-selectivity further emphasizes the necessity of rigorously quantifying the SI to ensure any potent anti-AMR lead compound spares healthy mammalian cells (Al-Sodany et al., 2018).

Finally, the layered defense strategy is also mediated by macromolecules; proteins from young leaves of *V. encelioides* exhibit moderate antimicrobial activity against various bacterial and fungal species, demonstrating a chemical and biological defense strategy (Kaur et al., 2022). The plant’s allocation strategy is predicted by the Limiting Resource Model (LRM), which suggests that when the host plant experiences stress (parasitism), it increases the allocation to shoot mass, directing more resources to the area where many of its chemical defenses (SLs) are sequestered, potentially suggesting a prioritized defense response to biotic stress (Evans and Borowicz, 2013).

### 5.3 Combating resistance: new approaches

The potential of the *Verbesina* genus to address resistance extends beyond direct antimicrobial activity, encompassing therapeutic strategies that support the host immune system, target chronic diseases, and leverage modern nanotechnology for enhanced efficacy. This multifaceted utility highlights the strategic value of the plant as a bioprospecting source for complex, next-generation scaffolds.

#### 5.3.1 Host-mediated and anti-inflammatory strategies

The established traditional use of *Verbesina* for wound healing and inflammation provides compelling *in vivo* evidence supporting its systemic anti-infective and anti-inflammatory potential. Topical application of the methanolic extract (VCME) of *V. crocata* to incisional wounds in a murine model demonstrated significant acceleration of the healing process, reducing the final closure time (FCT) to 12 days, which was faster than the positive control (Recoveron®) and the negative control (Vaseline). Crucially, the VCME significantly increased the tensile strength of the healed tissue (700 ± 148.3 g) compared to the positive control (640 ± 259.9 g), indicating superior collagen organization (García-Bores et al., 2020; Heyerdahl-Viau et al., 2023). Histological analysis confirmed that the extract advanced the healing process to the remodeling phase by promoting the synthesis of mature collagen and significantly reducing the number of fibroblasts. This efficacy is attributed to the extract’s anti-inflammatory action (linked to sitosterol glycoside and known sesquiterpenes) and its potential for immune modulation (phylloflavan activates macrophages), demonstrating the extract’s ability to control the initial infective and inflammatory phases of wound repair (García-Bores et al., 2020).

The precise structural requirement for this anti-inflammatory activity is defined by the core eudesmane scaffold’s functionalization. Pure 4β-cinnamoyloxy, 1β,3α-dihydroxyeudesm-7,8-ene demonstrated a notable anti-inflammatory activity, achieving 83.18% edema inhibition *in vivo* (ED_50_ = 0.43 μM). This effect is strongly dependent on the integrity of the cinnamate moiety: chemical modifications such as acetylation and hydrogenation resulted in a significant reduction in anti-inflammatory activity (up to 60% reduction for the hydrogenated derivative), underscoring a prevailing role of the unsaturated double bonds of the cinnamate scaffold in this biological effect (Dalla Via et al., 2015). This mechanism of reducing the inflammatory phase is further supported by the EO fraction, which demonstrated strong anti-inflammatory and antipyretic activities statistically similar to or exceeding commercial drugs like Dexamethasone and Dipyrone, respectively. The anti-inflammatory effect (carrageenan-induced peritonitis model) is associated with the reduction of the pro-inflammatory cytokines TNF-α (up to 73.52%) and IL-1β (up to 87.71%), consistent with the activity of its major components, β-caryophyllene and Germacrene D, which are potent inhibitors of pro-inflammatory cytokine production (Villaseñor and Ortiz, 2012).

This mechanism of host-defense enhancement is further validated by studies on *Verbesina oerstediana* Benth. [Asteraceae], which demonstrated crucial immunomodulatory activity. Extracts of *V. oerstediana* (VEOEBA) significantly enhance the antimicrobial efficacy of human monocytes and neutrophils against *S. aureus* infections. Mechanistically, this enhancement is attributed to the extract’s ability to significantly stimulate the production of Neutrophil Extracellular Traps (NETs). NETs are extracellular fibers composed of DNA-histone complexes that entrap and kill bacteria, representing a critical, non-phagocytic defense mechanism, suggesting that *Verbesina* compounds can serve as adjunctive AMR therapies by boosting the host’s innate immune response (Yaseen et al., 2017).

#### 5.3.2 Non-infectious therapeutic applications (chronic disease and SAR)

The chemical versatility of the genus ensures its therapeutic utility extends to high-value areas outside of acute infection, particularly in managing chronic human diseases. The guanidino-amide alkaloids are a significant class of potent hypotensive agents (Botta et al., 2003). The novel monomer caracasanamide from *V. caracasana* demonstrated a dose-dependent hypotensive effect, reducing mean blood pressure while simultaneously increasing cardiac inotropism (dP/dt) and respiratory function (Delle Monache et al., 1992; Monache et al., 1993; Corelli et al., 1996). Critically, this compound does not decrease cardiac inotropism or chronotropism at nontoxic doses, in contrast to most other hypotensive drugs tested, making it a clearly advantageous therapeutical aid for moderate arterial hypertension (Delle Monache et al., 1991; Monache et al., 1993). The pursuit of structural analogs, such as the dimeric caracasandiamide (Delle Monache,’ et al., 1996) and prenylagmatine analogs (Delle Monache et al., 1999; Carmignani et al., 2001), demonstrated that subtle variations within the alkaloid structure significantly enhance pharmacological action, providing a clear path for chemical modification and optimization of the genus’s cardiovascular agents (Compagnone et al., 2008; Corelli et al., 1996; Delle Monache et al., 1999).

Beyond cardiovascular agents, the genus demonstrates efficacy in managing metabolic syndrome. The extract of *Verbesina persicifolia* was found to significantly alter glucose tolerance, enhance glucose uptake in skeletal muscle, and notably inhibit glycogenolysis in the liver, suggesting it is effective in controlling blood glucose following a heavy carbohydrate diet (Perez et al., 1996b). Furthermore, the ethanolic extract of *V. encelioides* roots exhibited significant hypolipidemic activity in rats, resulting in a measured decrease in total cholesterol, triglycerides (TGL), and very low-density lipoproteins (VLDL), while maintaining or increasing high-density lipoproteins (HDL) (Banasthali University, 2017). The genus’s therapeutic scope is rapidly expanding to include neuroprotection and anticonvulsant activity; the methanolic extract of *V. persicifolia* demonstrated highly significant anticonvulsant activity in the zebrafish seizure model, resulting in a 100 survival rate after a PTZ-induced seizure event at a non-toxic concentration (López-Rosas et al., 2025). Aqueous extracts of *V. crocata* also exhibit measurable diuretic activity, comparable to the positive control furosemide in increasing sodium and potassium clearance and excretion (Heyerdahl-Viau et al., 2023).

The most compelling evidence for a favorable SI drived from a Verbesina species is provided by research on *V. lanata*. Compounds closely related to the genus’s characteristic scaffolds were evaluated for anti-protozoal activity against Leishmania major promastigotes, showing dose- and time-dependent activity with IC_50_ values ranging from 4.9 to 25.3 μM. Crucially, the toxicity against normal murine macrophages (CC_50_) ranged from 432.5 to 620.7 μM, yielding a high SI of up to 88.2 for Arbusculin Derivative ((1*R*,4S,4a*S*,7*R*)-1-methyl-7-(prop-1-en-2-yl)octahydro-2*H*-1,4a-(epoxymethano)naphthalen-4-yl acetate). This SI value is substantially better than that of the standard drug, meglumine antimoniate (glucantime), which had an SI of 40.0. This quantitative comparison provides definitive validation that eudesmane scaffolds can yield lead compounds with a high therapeutic window (Wu et al., 2024).

#### 5.3.3 Technological valorization and biocontrol applications

The chemical complexity of *Verbesina* allows its extracts to be leveraged in modern nanotechnology, offering a rapid, safe, and environmentally friendly avenue for combating AMR. The methanolic and ethanolic extracts of *V. encelioides* contain natural reducing agents (polyphenols, flavonoids, and terpenoids) capable of performing the green synthesis of silver nanoparticles (AgNPs). These fabricated AgNPs then exhibit superior antimicrobial action compared to the extracts alone against both bacteria (*E. coli* and *V. cholerae*) and fungi (*Aspergillus* spp.) (Bhati-Kushwaha and Malik, 2013; Bezerra et al., 2024). This successful synthesis is further supported by the use of purified saponin extracts from *V. encelioides* as natural reducing and stabilizing agents for AgNPs used in dermal applications, such as the management of acne (Verma et al., 2019). The successful synthesis using *V. crocata* further supports the viability of this strategy, with the synthesized Ag/AgCl-NP’s exhibiting sizes less than 100 nm, indicating strong potential for enhanced antimicrobial and anti-biofilm activity (Ramírez-Herrera et al., 2025). The broad utility of Verbesina metabolites suggests non-traditional applications in combating resistance, as phenolic and other compounds within the aqueous extract serve as natural reducing agents for the green synthesis of AgNPs, which exhibit enhanced antimicrobial activity against pathogens like *E. coli* and *V. cholerae*. This suggests Verbesina extracts can be directly leveraged in novel drug delivery and anti-AMR formulations (Kaur et al., 2022).

Furthermore, the plant’s aggressive ecological resilience is being valorized for biocontrol applications. The nematicidal capability of *Verbesina* opens a new avenue for AMR research by targeting biocontrol agents that reduce reliance on synthetic pesticides (Oka, 2012). Aqueous extracts of the plant show strong nematicidal activity against the root-knot nematode *M. javanica*, indicating its potential as a green manure or natural nematicide. Furthermore, the plant’s aggressive ecological resilience is being valorized for biocontrol applications. A patent claims an anti-termite herbal composition derived in part from the flowers and leaves of *V. encelioides*, demonstrating its commercial potential as an eco-friendly pesticide. This supports the strategy of leveraging the plant’s natural chemical defense system, driven by compounds like alkaloids and saponins, as a low-cost, sustainable biopesticide solution (Banasthali University, 2017).

The demonstrated allelopathic potential of *V. encelioides* offers an innovative route for biocide valorization as an environmentally friendly control agent for cyanobacterial blooms in eutrophic water bodies. The aqueous extract of the aerial parts of *V. encelioides* exhibits a strong anti-cyanobacterial effect on the growth of the harmful species, *Microcystis aeruginosa*, with a maximum Inhibitory Rate of 93% achieved at 1 mg/mL concentration. This efficacy is noted to be due to the compounds interfering with photosynthesis and ultimately inducing cell death (Lahlali et al., 2023).

The severe agricultural impact of *V. encelioides* as a weed and as a reservoir host for thrips (vectors for Tomato Spotted Wilt Virus, TSWV) strongly justifies the development of *Verbesina*-derived compounds as high-value, natural biopesticides (Grichar and Sestak, 1998; Grichar et al., 2012; Sharma et al., 2018). Beyond biopesticides, the genus exhibits potential for industrial valorization. Early screenings for renewable energy sources identified *V. alternifolia* as a rubber-producing species yielding a quantifiable percentage of hydrocarbon (0.4%), confirming its utility as a source of low-molecular-weight polyisoprenes and hydrocarbon feedstocks (Carr and Bagby, 1987). Conversely, the extracts demonstrate significant agricultural potential by acting as growth biostimulants, particularly in high-value crops like strawberry (*Fragaria ananassa*). Application of aqueous extracts of *V. sphaerocephala* and *Verbesina fastigiata B.L.Rob. and Greenm [Asteracea]* resulted in notable increases in productive parameters, including up to a 207.14% Agronomic Efficiency Index (IEA) for fruit and flower production (Velasco-Ramírez et al., 2022a). The intrinsic chemical resilience of *V. encelioides* also opens a high-value application in phytoremediation, suggesting its potential role in the stabilization or phytoextraction of heavy metals from contaminated mine waste (Carmona-Chit et al., 2016).

### 5.4 Computational validation of the cinnamoyloxy anchor: pre-covalent target engagement

While the α-methylene-γ-lactone is the established reactive warhead responsible for irreversible Michael addition, the non-covalent interactions immediately preceding this alkylation step dictate target specificity and overall thermodynamic stability. To structurally elucidate why the characteristic cinnamoyloxy ester is essential for high-potency bioactivity, a comparative molecular docking study was executed. The full eudesmane scaffold (Compound **1**) and its truncated parent core (Verbesindiol) were evaluated against two validated targets reflecting the genus’s therapeutic duality: human IKKβ (PDB: 4KIK), the primary upstream kinase regulator of the NF-κB inflammatory pathway, and wild-type MRSA PBP2a (PDB: 3ZG0).

The molecular docking simulations were performed to quantify the binding affinities of the primary eudesmane scaffolds within target active sites. Target crystal structures for human IKKβ (PDB: 4KIK) (Liu et al., 2013) and MRSA PBP2a (PDB: 3ZG0) (Otero et al., 2013) were retrieved from the RCSB Protein Data Bank. Protein preparation involved the removal of crystallographic water molecules, addition of polar hydrogens, and Gasteiger charge assignment. Ligand geometries for Compound **1** and Verbesindiol were optimized using the MM2 force field to ensure minimum energy conformations (Allinger, 1977). Docking was executed using a grid-based search algorithm with a search space centered on the co-crystallized ligand coordinates to encompass all relevant binding residues. Interaction profiles, including hydrogen bonding and π-driven contacts, were visualized and analyzed to determine the thermodynamic contribution of the cinnamoyloxy moiety. The protocol was validated by redocking co-crystallized ligands, achieving a highly accurate RMSD of 0.476 Å for 4KIK and 1.965 Å for 3ZG0. Detailed grid box dimensions, exhaustiveness parameters, and complete interaction matrices are provided in the Supplementary Material.

#### 5.4.1 Mammalian anti-inflammatory target (NF-κB pathway: IKKβ)

**FIGURE 5:**
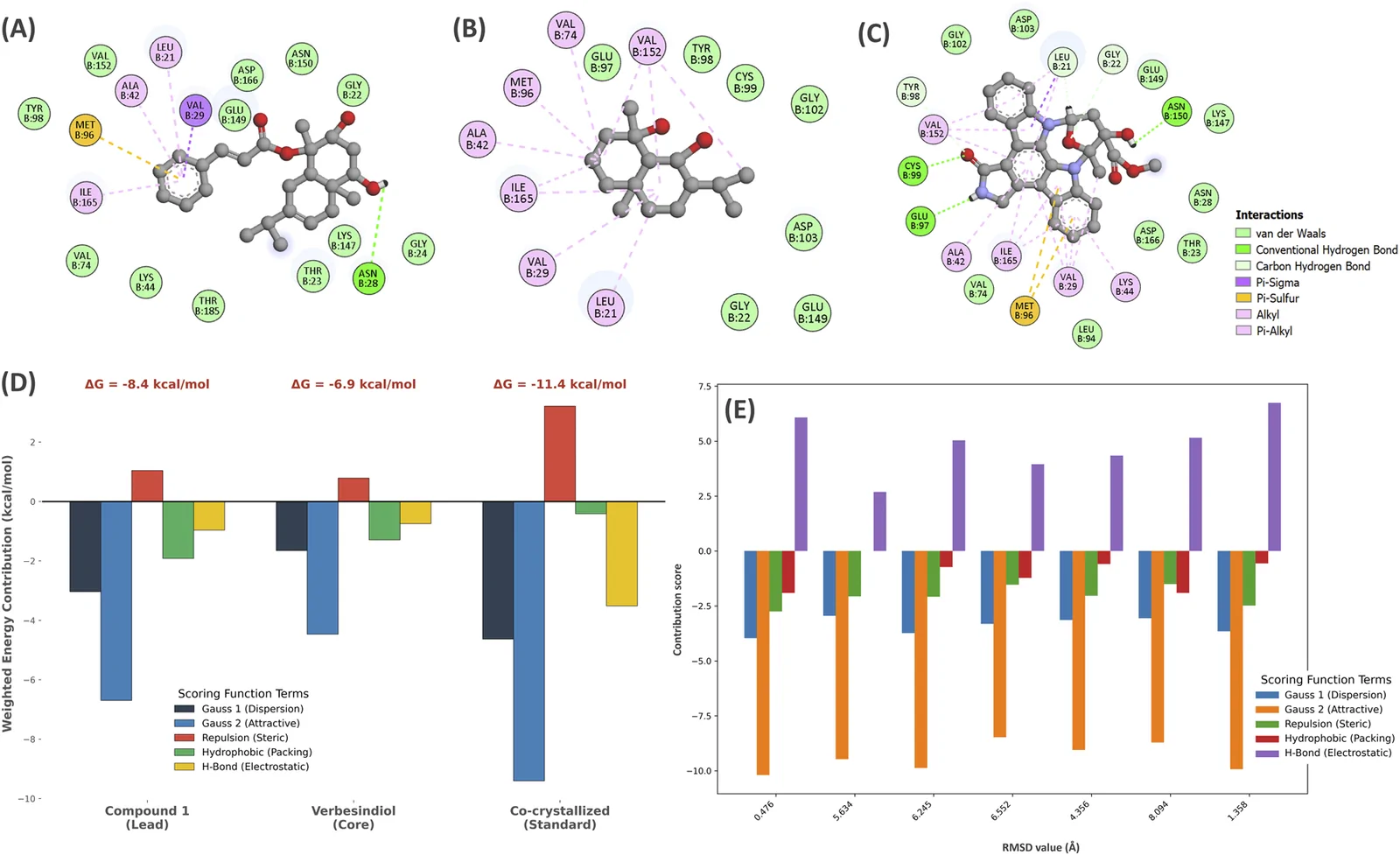
Structural and Thermodynamic Profile of IKKβ Target Engagement (PDB: 4KIK). **(A–C)** 2D interaction maps for **(A)** Compound **1**, **(B)** Verbesindiol, and **(C)** the co-crystallized standard. **(D)** Comparative weighted energy contributions (kcal/mol) for the three ligands. **(E)** Decomposition of scoring function terms across multiple docking poses for Compound **1**.

Docking against the IKKβ kinase domain revealed that the cinnamate ester acts as a critical structural anchor rather than passive lipophilic bulk. Compound **1** demonstrated robust target engagement (Figure 5A) with a binding affinity of ΔG = −8.4 kcal/mol (Figure 5B). As illustrated in the 2D interaction map (Figure 5A), the aromatic geometry of the ester exclusively drives high-value, non-covalent contacts, including a π-σ interaction with VAL29, π-sulfur bonding with MET96, and extensive π-alkyl packing with LEU21, ALA42, and ILE165. Furthermore, it facilitates a stabilizing hydrogen bond with ASN28. This structural fit is validated against the co-crystallized standard (Figure 5C), which shares overlapping binding coordinates.

Energetic fingerprinting of the binding pocket confirms that this high affinity is thermodynamically driven by superior attractive forces (Figure 5D). In Compound **1**, the attractive dispersion (gauss_2) contribution is −6.698 kcal/mol, and hydrophobic packing (hydrophobic_4) contributes −1.918 kcal/mol. These values align closely with the energetic profile of the co-crystallized standard, validating the pharmacological relevance of the cinnamoyloxy-eudesmane template.

In stark contrast, *in silico* removal of the ester to yield the parent Verbesindiol core resulted in a severe collapse in binding affinity to ΔG = −6.9 kcal/mol. The truncated core entirely lost the stabilizing hydrogen bond and the critical π-σ interaction. This is reflected in the energetic decomposition (Figure 5D), where the attractive dispersion (gauss_2) falls to −4.471 kcal/mol and hydrophobic packing is reduced to −1.293 kcal/mol.

The resulting quantitative ΔG penalty of +1.5 kcal/mol proves that the cinnamate moiety is essential for firmly positioning and locking the eudesmane scaffold within the NF-κB regulatory pathway. By maximizing π-driven non-covalent bonding, the ester provides the necessary thermodynamic stability to pre-align the α-methylene-γ-lactone warhead for subsequent nucleophilic attack. This docking protocol was internally validated by redocking the co-crystallized ligand with a highly accurate RMSD of 0.476 Å, while the conformational scoring stability across multiple poses (Figure 5E) confirms the structural robustness of the identified binding mode.

#### 5.4.2 Bacterial target (MRSA PBP2a)

**FIGURE 6:**
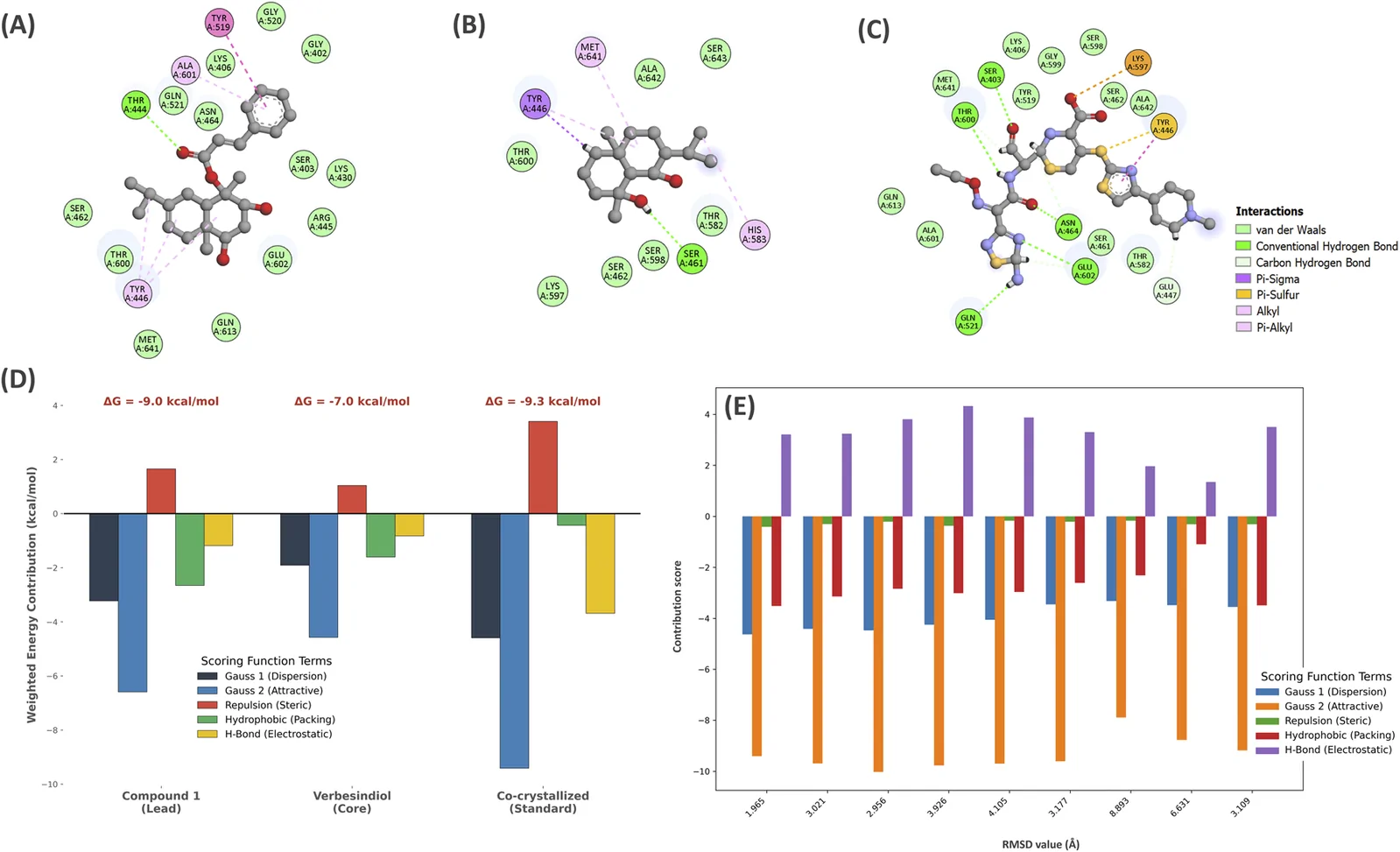
Structural and Thermodynamic Profile of MRSA PBP2a Target Engagement (PDB: 3ZG0). **(A–C)** 2D interaction maps for **(A)** Compound **1**, **(B)** Verbesindiol, and **(C)** the co-crystallized standard. **(D)** Comparative weighted energy contributions (kcal/mol) for the three ligands, highlighting the hydrophobic and attractive dispersion advantages of the lead scaffold. **(E)** Decomposition of scoring function terms across multiple docking poses for Compound **1**, validating the stability of the primary binding model.

The aromatic anchoring mechanism observed in the mammalian models is heavily conserved when evaluated against the antimicrobial target. Against MRSA PBP2a, Compound **1** achieved an exceptional binding affinity of ΔG = −9.0 kcal/mol, nearly matching the highly optimized co-crystallized inhibitor (ΔG = −9.3 kcal/mol). The 2D interaction map (Figure 6A) illuminates the specific structural advantage of the cinnamate ester: it facilitates a crucial π-π T-shaped stabilization with TYR519 and strong π-alkyl interactions with TYR446 and ALA601. These interactions firmly lock the eudesmane scaffold within the active site cleft, providing the necessary spatial orientation for the α-methylene-γ-lactone warhead.

Thermodynamic fingerprinting (Figure 6D) confirms that this high potency is driven by a superior energetic fit compared to the parent core. In Compound **1**, the attractive dispersion (gauss_2) contribution is −6.593 kcal/mol, and the hydrophobic packing (hydrophobic_4) is remarkably high at −2.654 kcal/mol. This profile closely mirrors the “gold standard” energetic distribution of the co-crystallized ligand (Figures 6C,D), particularly in the dominant attractive dispersion and dispersion terms.

Conversely, the Verbesindiol core yielded a significantly weaker affinity of ΔG = −7.0 kcal/mol. Lacking the spatial reach and π-electron density of the cinnamate group, the diol failed to engage TYR519 (Figure 6B) and lost critical van der Waals networking across the binding pocket. The energetic decomposition (Figure 6D) quantifies this failure, with attractive dispersion falling to −4.574 kcal/mol and hydrophobic packing reduced to −1.604 kcal/mol. This +2.0 kcal/mol penalty reinforces the conclusion that the cinnamoyloxy anchor is thermodynamically indispensable for high-potency anti-AMR activity. Consistent with the mammalian target, the structural robustness of this binding mode is supported by the conformational scoring stability observed across multiple low-RMSD poses (Figure 6E).

#### 5.4.3 Conclusion on pharmacophore optimization

These comparative computational results address a fundamental mechanistic query regarding the eudesmane library. The cinnamoyloxy ester improves binding affinity far beyond the baseline parameters of the simple alkylating lactone core. By maximizing π-driven non-covalent bonding, resulting in ΔG improvements of −1.5 to −2.0 kcal/mol, the ester acts as a potent thermodynamic driver. As summarized in Table 2, the ester stabilizes the pre-reaction complex by significantly increasing attractive dispersion (gauss_2) and hydrophobic packing (hydrophobic_4). This interaction severely restricts the ligand’s conformational freedom and perfectly aligns the α-methylene-γ-lactone warhead for subsequent nucleophilic attack by target residues.

**TABLE 2:** Comparative binding affinities and interaction profiles of verbesina scaffolds.

Target (PDB)	Ligand	Binding affinity (ΔG kcal/mol)	Key interactions
MRSA PBP2a (3ZG0)	Compound **1**	−9	H-bond: THR444; π-π T-shaped: TYR519; π-Alkyl: TYR446, ALA601
	Verbesindiol	−7	H-bond: SER461; π-σ: TYR446; Alkyl: MET641
	Co-crystallized	−9.3	H-bond: SER403, THR600, ASN464, GLU602, GLN521
Human IKKβ (4KIK)	Compound **1**	−8.4	H-bond: ASN28; π-σ: VAL29; π-Sulfur: MET96
	Verbesindiol	−6.9	π-Alkyl: VAL74, VAL152, VAL29, LEU21, ILE165
	Co-crystallized	−11.4	H-bond: CYS99, GLU97, ASN150; π-σ: VAL152

ΔG represents the estimated free energy of binding. Weighted energy terms (gauss_2: attractive dispersion; hydrophobic_4: hydrophobic packing) are calculated using the smina scoring function. RMSD values indicate the redocking accuracy of the co-crystallized ligand to validate the docking protocol. All calculations were performed using an exhaustiveness of 8 and a 0.476 Å RMSD success threshold.

## 6 Critical analysis: the therapeutic index and drug feasibility

The successful translation of a natural product into a pharmaceutical lead depends entirely on its ability to demonstrate preferential toxicity, which is quantified by the Selectivity Index (SI = IC_50_/MIC) (Indrayanto et al., 2021b). The inherent chemical characteristic of the *Verbesina* genus, defined by highly reactive SLs and potent alkaloids, presents a formidable challenge: the strong potential for efficacy (MIC) is inextricably linked to the risk of host toxicity (IC_50_). This section critically analyzes the compiled data, providing quantitative and structural arguments to assess the feasibility of isolating and optimizing SL scaffolds with an intrinsically superior therapeutic window.

### 6.1 Calculation and interpretation of the selectivity index (SI)

**FIGURE 7:**
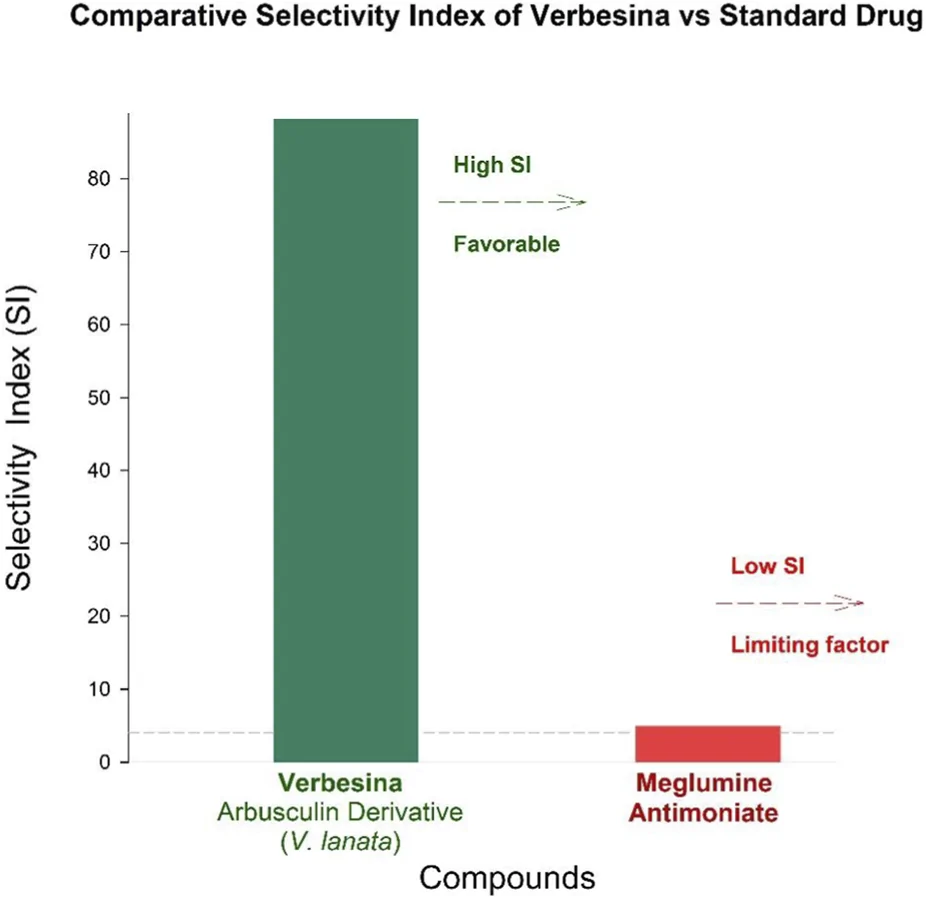
Translational feasibility assessed by selectivity index. Comparison of selectivity index (SI) values shows that the Verbesina arbusculin derivative from V. lanata exhibits a markedly higher SI than that of meglumine antimoniate, indicating a more favorable balance between antiparasitic activity and host-cell toxicity. This contrast highlights the stronger translational potential of the Verbesina-derived scaffold.

The determination of the SI is complicated by the presence of a diverse chemical arsenal, yet quantitative evidence confirms that a favorable SI is achievable through targeted isolation (Figure 7; Table 3). In the context of natural product bioprospecting, a Selectivity Index (SI) value ≥ 10 is widely accepted as the minimum threshold for identifying a promising “hit” against microbial or protozoal targets. Scaffolds achieving an SI > 50 are considered high-priority leads with a wide therapeutic window, as they demonstrate a significant margin of safety between the effective antimicrobial dose and host-cell toxicity (CC_50_).

**TABLE 3:** Comparative analysis of selectivity index (SI) and efficacy-toxicity duality for verbesina metabolites and extracts.

Category	Compound/Extract	Efficacy (target and value)	Toxicity (cell line and value)	Selectivity index (SI)	References
I. Confirmed high selectivity (SI >> 1)					
Anti-Protozoal Lead	Arbusculin Derivative (*V. lanata*)	*L. major* amastigotes 4.9–25.3 μM	NMM 432.5–620.7 μM	Up to 88.2	Wu et al. (2006a)
Non-Cytotoxic Triterpenes	Pseudotaraxasterol Derivatives (from *V. encelioides*)	Protozoa (Antiprotozoal activity) 32.2–48.2 μg/mL	HELF, (MRC-5) (CC_50_), > 64 μg/mL	>1.3–2.0	Ezzat et al. (2017)
Host-Immune Booster	*V. oerstediana* Acetone Bark Extract	*S. aureus*, significantly boosts killing at 10 μg/mL	Neutrophils (Viability), Non-cytotoxic at 10 μg/mL	High (Qualitative)	Albuquerque et al. (2006), Yaseen et al. (2017)
II. Efficacy and duality challenges					
Efficacy Benchmark (Germacrene)	Germacrene SL (*V. negrensis*)	*S. aureus*/*E. faecalis* (MIC), 64 μg/mL	CC_50_ on non-cancerous line: >64 μg/mL	≈1 (Implied)	Mora et al. (2013b)
Duality Case (Mild Uncoupler)	CDE (from *V. persicifolia*)	Undocumented	RLM (C_eff_), 20–100 μM (Mild Uncoupling)	≈1 (Implied)	Dalla Via et al. (2014)
Cytotoxicity Benchmark (Extract)	*V. turbacensis* Hydroethanolic Extract	(Low Activity assumed)	HepG2 cells (IC_50_), 334.6 μg/mL	High (Qualitative)	Caballero-Gallardo et al. (2023a)
Toxicity Benchmark (Isolated Toxin)	Galegine (Alkaloid) (from *V. encelioides*)	(Toxicity focus)	Hep G2 cells (IC_50_), 10.9 μg/mL	Very low SI (Implied)	Abbas et al. (2016)
Extract Duality (Allelopathy)	*V. encelioides* Root Leachate	Inhibition (Full Strength), −25.7% (RRG)	Stimulation (Low dilution), +36.5% (RRG)	Concentration-dependent	Inderjit et al. (2000a)

SI: Selectivity Index (CC_50_/IC_50_ or CC_50_/MIC); NMM: normal murine macrophages; HELF: human embryonic lung fibroblasts; RLM: rat liver mitochondria; RRG: radish root growth; IC_50_: standard inhibitory, MIC: minimum inhibitory constant; CC_50_: Cytotoxicity; GI_50_: growth inhibition; The SI for the Germacrene Efficacy Benchmark (MIC, 64 μg/mL) is inferred (≥1) as a specific non-cytotoxicity CC_50_ is currently undocumented, although correlated non-toxic triterpenoid CC_50_ values for *V. encelioides* exceed this concentration; Arbusculin Derivative: (1*R*,4*S*,4a*S*,7*R*)-1-methyl-7-(prop-1-en-2-yl)octahydro-2*H*-1,4a-(epoxymethano)naphthalen-4-yl acetate; Germacrene SL: (1*S*,6*R*,7*S*,10*S*,*Z*)-6-hydroxy-10-isopropyl-7-methoxy-3,7-dimethyl-4-oxocyclodec-2-en-1-yl cinnamate; CDE: (4β-cinnamoyloxy- 1β,3α-dihydroxyeudesm-7,8-ene).

The *Verbesina* genus provides several candidates that meet or exceed these clinical benchmarks. Specifically, the Arbusculin derivative from *V. lanata* achieves an SI of 88.2. This value is not only twice the required threshold for high priority leads but also significantly outperforms the standard anti-protozoal drug, meglumine antimoniate, which possesses a more restrictive SI of 40.0. Such quantitative differentiation provides the definitive validation that eudesmane scaffolds can yield lead compounds with a superior therapeutic window compared to existing synthetic or semi-synthetic options.

#### 6.1.1 Quantitative evidence for a favorable selectivity index

The most compelling quantitative evidence for a favorable SI derived from a *Verbesina* species is provided by research on *V. lanata*. This study evaluated compounds closely related to the genus’s characteristic eudesmane scaffolds for anti-protozoal activity against *Leishmania major* promastigotes, yielding IC_50_ values ranging from 4.9 to 25.3 μM. Crucially, the toxicity against normal murine macrophages (CC_50_) ranged from 432.5 to 620.7 μM. This differential resulted in a high SI of up to 88.2 for Arbusculin Derivative (Wu et al., 2006). This SI value is substantially better than that of the standard drug, meglumine antimoniate (glucantime), which had an SI of 40.0. This quantitative comparison provides definitive validation that eudesmane scaffolds can yield lead compounds with a high therapeutic window (Wu et al., 2024).

This potential for selectivity is also demonstrated qualitatively in other fractions. The EO of *V. macrophylla* provides a strong qualitative argument for a favorable SI. The EO is toxicologically safe (low hemolysis in human cells and LD_50_ > 5,000 mg/kg) while simultaneously exhibiting potent antimicrobial activity (MIC 16 μg/mL against *K. pneumoniae* and MIC 4 μg/mL against *C. albicans*). The low toxicity is correlated with the known safety profile of its major components. The finding that the essential oil is a potent antimicrobial and anti-inflammatory agent with a wide toxicological safety window validates the ethnopharmacological use and suggests that the essential oil of *V. macrophylla* may be a basis for developing drugs with natural anti-AMR effects (de Veras et al., 2021). Similarly, strong trypanocidal activity (up to 100% elimination at 200 μg/mL) against *T. cruzi* combined with the confirmed toxicological safety of the essential oil of the same species strongly indicates the potential for a favorable SI (Bezerra et al., 2024). Furthermore, the measured MIC of 64 μg/mL for a pure isolated SL ((1*S*,6*R*,7*S*,10*S*,*Z*)-6-hydroxy-10-isopropyl-7-methoxy-3,7-dimethyl-4-oxocyclodec-2-en-1-yl cinnamate) against key Gram-positive pathogens provides a critical benchmark for the efficacy component of the SI calculation (Mora et al., 2013). This further justifies the mandatory strategy of purification and structural investigation to identify and ultimately modify these powerful lead molecules (Mora et al., 2013; Ramseyer et al., 2017). This mechanism of host-defense enhancement is further validated by studies on *V. oerstediana*, which demonstrated crucial immunomodulatory activity by stimulating NET release without significantly affecting the viability of the neutrophils (viable NETosis), suggesting an ideal pharmacological profile for developing adjunctive AMR therapies (Yaseen et al., 2017).

#### 6.1.2 The duality challenge and evidence for mitigation

The core translational challenge for *Verbesina* SLs is their non-selective reactivity (Dalla Via et al., 2014). Experimental data confirms the characteristic duality of efficacy and potential toxicity inherent to the genus’s metabolites. A critical assessment of the *Verbesina* chemical library reveals that several potent metabolites possess a dangerously narrow therapeutic window, where the SI approaches 1 (Table 2). For example, the Germacrene SL from *V. negrensis* exhibits reliable antibacterial activity (MIC = 64 μg/mL), yet its lack of documented host-cell safety suggests a high risk of non-selective alkylation. Similarly, the guanidine alkaloid galegine demonstrates a clear translational limitation, with a high cytotoxicity (IC_50_ = 10.9 μg/mL) against HepG2 cells that correlate with its documented lethality in livestock. These low-SI compounds are categorized here as toxicological benchmarks rather than therapeutic leads.

The presence of these benchmarks underscores the necessity of identifying scaffolds that mitigate duality through structural “shielding.” Studies provide key evidence of dose-dependent biological activity; for instance, full-strength root leachate significantly suppressed radish seedling growth, whereas a 1:4 dilution promoted growth by up to +36.5% (Inderjit et al., 2000). This duality confirms the complex task of optimizing the therapeutic window, as the same extract contains compounds that can both severely inhibit and promote growth, demanding precise chemical isolation to separate high-priority leads from systemic toxins (Inderjit et al., 2000; Velasco-Ramírez et al., 2022b). This high performance is fundamentally linked to the extract’s dual nature: acting as allelopathic agents that inhibit seed germination, but also as potent biostimulants that significantly enhance crop growth and yield (Hernández-Pérez et al., 2025). Furthermore, the prominent antioxidant activity of the extracts, with the ethyl acetate fraction showing high capacity in assays like Ferric Reducing Power, reinforces the conclusion that the total efficacy profile of Verbesina relies on the complex synergistic interaction between potent (toxic) primary metabolites (like SLs and galegine) and high-concentration support molecules (like phenolic acids and flavonoids), necessitating the targeted isolation and study of all classes for optimal therapeutic outcomes (Rodríguez-Valdovinos et al., 2024).

Quantitative data models this separation challenge. Potency is concentrated in the crude extract and the ethyl acetate fraction, often considered the primary source of SLs (Gorja and Bandla, 2024). This finding is supported by the comparison demonstrating that the ethanolic extract (VE) is twice as potent as the aqueous extract (VA). The greater efficacy of the VE strongly suggests that the core bioactive agents responsible for the observed potent ROS-mediated apoptosis are concentrated within the non-polar fraction (Farshori, 2021). Therefore, optimizing the therapeutic window requires separating the potent efficacy-driving compounds (likely the highly reactive SLs) from the toxicity-mitigating or synergistic non-SL components (like the less-toxic triterpenoids and β-sitosterol glycosides) (Ezzat et al., 2017). This process minimizes the co-extraction of inactive, highly polar compounds and potentially concentrates the therapeutic molecules away from water-soluble toxins (like the alkaloid galegine), thereby optimizing the input material for subsequent isolation and structural modification aimed at generating a more favorable SI (Farshori, 2021). The severity of the SI challenge is mitigated by the isolation of less-toxic fractions (Ezzat et al., 2017). The low cytotoxicity found for the hydroethanolic *V. turbacensis* extract (IC_50_ of 334.6 μg/mL on HepG2) is a critical component for the future calculation of its SI. A high IC_50_ against a generalized cancer cell line, when compared to a low MIC, would yield a favorable SI, suggesting a low host toxicity (Caballero-Gallardo et al., 2023). Furthermore, the study on CDE from *Verbesina persicifolia* introduces a compelling argument for the genus’s potential selectivity, classifying CDE as a “mild” uncoupler. The authors stress that since *V. persicifolia* is widely used in Mexican traditional medicine for various complaints, administering CDE should not have dangerous toxic side-effects (Dalla Via et al., 2014). This high level of tolerability, coupled with robust efficacy at significantly lower doses (5 mg/kg and 10 mg/kg), strongly supports the potential for a favorable SI for the extract’s neuroprotective properties (Verma et al., 2025).

#### 6.1.3 The need for rigorous validation

The overall analysis reinforces the contemporary and direct validation for the central objective of the review: to rigorously compile the necessary IC_50_ data and calculate the SI to bridge the gap between proven high efficacy and unassessed safety (Ramseyer et al., 2017). The inherent allelopathic/phytotoxic property of the raw *Verbesina* extract is confirmed, which directly relates to the central challenge of the SI (Ramírez-Herrera et al., 2025). The strong *in vivo* wound healing performance of *V. crocata* suggests that the complex chemical mixture provides a favorable overall therapeutic profile, successfully controlling the inflammatory phase while promoting organized tissue remodeling (García-Bores et al., 2020). The successful valorization of *Verbesina* requires isolating the beneficial compounds from the potential toxins (like the guanidine alkaloid, galegine) to ensure its role as a safe, sustainable biostimulants or as a high-SI therapeutic agent (Hernández-Pérez et al., 2025).

### 6.2 Structure-activity relationship (SAR)

The establishment of reliable SAR for *Verbesina* metabolites is the indispensable prerequisite for designing less toxic, highly selective drug candidates with a favorable SI. This process meticulously links the complex C_15_ scaffold and its functional groups to the observed dual cytotoxic (IC_50_) and antimicrobial (MIC) activities.

#### 6.2.1 Foundational structural requirements and stereochemical precision

SAR metrics are dependent on unequivocal structural gold standards, which must resolve the complex stereochemistry and conformation of the bicyclic core. Errors in stereochemistry directly translate to flawed SAR conclusions regarding which compounds possess the most favorable therapeutic index (Jakupovic et al., 1987; Ramseyer et al., 2017).

The *cis*-Decalin Core: Early structural studies confirmed the complex stereochemistry of the *Verbesina* sesquiterpenes. The Verbesinols were found to be stereomeric with junenol, establishing the complex, rigid *cis*-ring juncture of the verbesinol core (Gardner et al., 1961). The X-ray confirmation of Verbesindiol’s structure was vital because it provided the definitive assignment of the *cis*-ring fusion and the stereochemistry of C-4 and C-6, correcting earlier ambiguous interpretations (Herz et al., 1982). Without this foundational structural evidence, pharmacological data would be difficult to reliably map to the true molecular scaffold. This rigorous structural confirmation is a necessary precursor to conducting meaningful SAR analysis and correlating the presence of the phenolic ester moiety with the observed acute cytotoxicity (IC_50_) or anti-AMR efficacy (MIC) (Xu et al., 2010).

Stereochemical Corrections and Analysis: Establishing a rigorous SAR requires painstaking chemical elucidation. The definitive structure of Rupestrol was established through a series of chemical conversions, including acetylation, hydrolysis, and periodate oxidation, demonstrating the complexity required for rigorous SAR determination (Box et al., 1977). The use of Nuclear Overhauser Effect (NOE) experiments validated the configuration of the C-4 hydroxyl group in Verbesindiol derivatives, confirming the original assignment and contradicting proposed changes by other research groups (Banerjee et al., 1985). ^1^H-NMR spectroscopy corrected earlier assignments for α- and β-Verbesinol esters by measuring very small coupling constants (J_5,6_ and J_6,7_), establishing that the compounds likely adopt absolute configurations where the C-7β-configuration of the isopropyl group is consistently observed [97] (Bohlmann and Zdero, 1979). The absolute configuration of Rupestrinol was determined via the Circular Dichroism (CD) curve of its derived ketone, suggesting it has the same absolute configuration as the parent compound Rupestrol (Box et al., 1977). The precise stereochemical determination, which confirmed the C-6 hydroxyl and C-10 methyl groups were cis and axial, is foundational for establishing accurate SAR metrics, as this axial arrangement dictates the spatial presentation of the C-6 cinnamate ester pharmacophore (Herz and Kumar 1981).

Conformational Flexibility: The X-ray analysis of C-4 cinnamates confirmed the existence of two crystallographically independent molecules in the crystal, differing only in the conformations of the side chains, highlighting the inherent conformational flexibility of the molecule that can impact its biological interaction (Martinez et al., 1983). The complexity of structural diversity in eudesmane derivatives can be explained by the germacrene precursor existing in different conformations, which ultimately results in eudesmane derivatives with different configurations (Jakupovic et al., 1987). Similarly, precise SAR analysis in the Elemanolide series (Zempoaline C) required definitive *X*-ray analysis due to their conformational flexibility (Ortega et al., 1985). The successful characterization of V. turbacensis eudesmanes employed two-dimensional NMR experiments, including COSY, NOESY, and HMBC, to achieve detailed structural and stereochemical assignments (Amaro-Luis et al., 2002). Furthermore, the *X*-ray determination of Verocephol’s structure provided the necessary resolution for accurately interpreting the molecule’s inherent reactivity to its observed activities (Salmon et al., 1985).

#### 6.2.2 Functional group dependence and mechanistic shift

The structural diversity of the SLs, including highly oxygenated enantio-eudesmanes like Rupestrol (Box et al., 1971) and novel cadinene-type SLs (Arciniegas et al., 2020), demonstrates that the core cytotoxic and antimicrobial action is highly dependent on functional group placement (Wu et al., 2006). This precise structural work is the prerequisite for comparing the efficacy (MIC) and cytotoxicity (IC_50_) of different scaffolds to achieve a favorable SI (Bohlmann and Lonitz, 1978).

The Alkylating Pharmacophore: The potential for any SL to succeed as a therapeutic agent is intimately linked to the highly reactive α-methylene-γ-lactone moiety, which acts as a template for non-reversible alkylation. The persistent co-occurrence of the cinnamate ester (the α,β-unsaturated moiety that acts as the electrophile) with other terpenoids reinforces the hypothesis that the genus’s potent effects are driven by these functionalized compounds (Bohlmann et al., 1982).

Hydroxyls, Acetyls, and Mechanism: A pivotal structure-activity study on derivatives of Compound 1 revealed that subtle structural modifications drastically alter the cellular target, even when GI_50_ values remain comparable (Dalla Via et al., 2015). Native Hydroxyls (Compound **1** and **3**, Figure 4) are crucial for inducing Ca^2+^-dependent Mitochondrial Permeability Transition (MPT). This effect is preventable by MPT inhibitors, supporting the role of the hydroxyl groups in MPT induction. Conversely, masking the hydroxyl groups with Acetate Substituents (Compound **2** and **4**, Figure 4) shifts the mechanism dramatically. These acetylated derivatives cause injury primarily by directly Inhibiting Complex II (Succinate dehydrogenase) of the mitochondrial respiratory chain and inducing an uncoupling effect on oxidative phosphorylation.

Structural Features for Activity: The biological activity of eudesmane sesquiterpenoids is strongly determined by the types, positions, and quantity of substituent groups. Anti-inflammatory activity, for instance, is significantly influenced by substitutions at pivotal positions like C-8, C-11, and C-13. An increase in the number of hydroxy substituents often correlates with an enhancement in anti-inflammatory efficacy. Furthermore, the terminal double bond between C-4 and C-15 (Δ^4(15)^ double bond) could significantly enhance this activity (Wu et al., 2024).

#### 6.2.3 SAR challenges and translational optimization

The successful application of SAR must account for the genus’s chemical complexity and the translational necessity of reducing toxicity (Kaur et al., 2021).

Targeting Alkaloid SAR for Analogue Design: Design principles established for the guanidino-amide alkaloids offer a parallel path for SL modification efforts. For hypotensive activity in this class, the presence of the double bond was found to be of major importance, while the absence of methoxy groups on the aromatic ring resulted in a measurable increase in hypotensive activity (Corelli et al., 1996).

Translational Strategy: A major strategy for optimizing the SI is the development of structural analogues aimed at reducing toxicity while enhancing effectiveness. This involves generating monofunctional alkylants or using chemical modifications to introduce substituents that alter the activity and safety profile (Kaur et al., 2021).

The Absolute Requirement: The initial step requires establishing a reliable SI for potent SLs, which is only meaningful after meticulous chemical elucidation provides the unequivocal structural gold standard (Ramseyer et al., 2017). Critically, the determination of the exact stereochemistry for the eudesmane derivatives from V. turbacensis provides necessary benchmarks, as correlations drawn between potency (MIC/IC_50_) and subtle structural differences would be scientifically unfounded without this foundational crystallographic work (Bruno-Colmenárez et al., 2010). The successful assembly of the 152,050 bp plastome of *V. alternifolia* provides a crucial genetic reference point that can help minimize the impact of geographical and chemotypic variability, supporting the goal of standardization necessary for reproducible SAR studies (Tomasello et al., 2024).

#### 6.2.4 Quantitative mapping of scaffolds and chemical functionalization

The translation of *Verbesina* chemistry from wild botanical defense networks into high-selectivity anti-AMR leads is structurally governed by three core chemical features. Deconstructing these structural elements reveals how chemical modifications act as precise molecular “dials” to shift antimicrobial potency (MIC), mammalian cytotoxicity (IC_50_), and the resulting SI.

The fused, unsaturated lactone core serves as the primary driver of non-reversible, covalent alkylation via Michael-type addition with cellular sulfhydryl groups. When this lactone warhead is present and sterically unshielded (as seen in classic elemanolide and germacranolide scaffolds), the molecule exhibits high, broad-spectrum antiproliferative and antimicrobial activity. However, this unchecked electrophilic reactivity often compromises cellular specificity, dragging the Selectivity Index toward unity (SI ≈ 1). Conversely, in scaffolds where the classic lactone ring is absent, such as the parent diol *Verbesindiol* or specific *Arbusculin* derivatives, the mode of action shifts entirely away from non-selective, systemic alkylation toward structure-specific target binding pockets. This structural transition preserves therapeutic efficacy while drastically reducing host-cell toxicity (CC_50_ > 400 μM), lifting the safety window to a highly favorable index (SI = 88.2).

The polyhydroxyl layout around the bicyclic *cis*-decalin skeleton dictates the molecule’s overall hydrophilic-lipophilic balance (HLB) and controls target engagement. An increase in the density of native, exposed hydroxyl substituents (exemplified by the pentahydroxy architecture of *Rupestrol* and the triol frameworks of *V. virgata*) provides multiple hydrogen-bond donor and acceptor sites. In mammalian targets, these unmasked hydroxyl groups are highly essential for inducing Ca^2+^-dependent Mitochondrial Permeability Transition (MPT), causing rapid mitochondrial swelling and subsequent apoptosis. From an SAR perspective, tuning the number and spatial orientation of these hydroxyl groups serves to modulate baseline host-cell clearance (CC_50_) without disrupting the fundamental antimicrobial template.

The conjugation of a lipophilic, aromatic cinnamoyloxy or *p*-coumarate ester to the eudesmane core acts as an indispensable structural booster for pharmacological potency. As validated by comparative computational modeling, the structural bulk of the cinnamate arm does not function as passive lipophilic weight; instead, its distributed π-electron cloud drives high-value, non-covalent target engagement (including critical π-π T-shaped stacking with TYR519 and π-σ packing with VAL29). This aromatic lock provides a substantial thermodynamic binding advantage (ΔΔG = −1.5–2.0 kcal/mol) that firmly pre-aligns and stabilizes the chemical scaffold inside pathogenic binding clefts prior to any downstream reaction. Consequently, cinnamoyl esterification acts as a primary structural prerequisite for achieving high-potency antimicrobial profiles (such as 6β-cinnamoyloxy-3β,4α-dihydroxyeudesmane achieving an MIC_100_ of 3.9 μg/mL), making the cinnamate anchor a fundamental requirement for optimizing clinical selectivity windows.

## 7 Discussion and translational direction

The data compiled within this review strongly validate the ethnopharmacological use of the *Verbesina* genus, confirming that its specialized metabolites, particularly the Sesquiterpene Lactones (SLs), provide a high-potency chemical arsenal against Antimicrobial Resistance (AMR). However, the inherent duality of efficacy and host toxicity, driven by the reactive α-methylene-γ-lactone pharmacophore defines the core translational challenge. Moving beyond promising MIC and IC_50_ data requires a rigorous roadmap focused on maximizing the Selectivity Index (SI) through chemical optimization, standardization, and targeted bioassay-guided fractionation.

### 7.1 The imperative for chemical optimization and analogue development

The SAR analysis (Section 5.2) demonstrated that subtle changes in the SL scaffold, such as masking hydroxyl groups with acetate substituents can drastically shift the cellular mechanism, from inducing Mitochondrial Permeability Transition (MPT) to inhibiting respiratory Complex II (Dalla Via et al., 2015). This complexity confirms that simple isolation of the natural product is insufficient; future success hinges on advanced medicinal chemistry to modify and detoxify the highly active natural scaffolds.

Mitigating Toxicity via Semi-Synthesis: The primary goal is to mask the non-selective reactivity of the α-methylene-γ-lactone core while preserving antimicrobial efficacy. The most promising strategy is the semi-synthesis of structural analogues aimed at reducing general cytotoxicity. Only compounds with a rigorously confirmed favorable SI can proceed toward this chemical optimization.

Synthetic Access and Scale-Up: For many valuable but scarce metabolites, such as the benzofuran-3-one derivative from *V. luetzelburgii* natural abundance is too low for comprehensive *in vivo* and SI testing, underscoring the necessity of a robust synthetic organic chemistry program (Pergomet et al., 2017). This capability is also critical for the scale-up and optimization of the guanidino-amide alkaloid scaffolds, as evidenced by the successful total synthesis of hypotensive analogs (Carmignani et al., 2001; Botta et al., 2003).

### 7.2 Standardization and bioassay-guided isolation

Translational research on Verbesina metabolites is complicated by high natural variability, requiring strict control over raw material quality and isolation protocols. Comparative analysis of reported IC_50_ and MIC values reveals discrepancies that are primarily driven by extraction methodology and solvent polarity. For instance, ethanolic extracts of *V. encelioides* (IC_50_ = 310 μg/mL) demonstrate twice the potency of aqueous extracts (IC_60_ = 650 μg/mL), confirming that the strategic concentration of lipophilic sesquiterpene lactones is essential for reproducible bioactivity (Farshori, 2021). To transition from preliminary screenings to standardized pharmacological benchmarks, the following strategic pillars must be addressed:

1. Standardization of Chemotypes: The measured IC_50_ and MIC values are susceptible to ontogenetic and geographical chemical variation, as the relative percentage of key sesquiterpenes can fluctuate significantly depending on the plant’s maturity (Albuquerque et al., 2006). Future work must implement high-resolution analytical standards like HPLC profiling to define standardized chemotypes and minimize this variability, thereby ensuring the reproducibility of the SI calculation.
2. Genetic Anchoring: The successful assembly of the complete plastome for *V. alternifolia* provides a crucial genetic reference point, which should be leveraged to map and minimize the impact of genetic and chemotypic variability when working with new accessions (Tomasello et al., 2024).
3. Targeted Toxicity Assessment: Rigorous SI calculation requires toxicity profiles (IC_50_ on non-cancerous lines) for every potent isolate to bridge the gap between high efficacy and unassessed safety (Ramseyer et al., 2017). Furthermore, the scarcity of information on many ethnobotanically important species, such as *V. crocata*, demands comprehensive characterization of active principles and toxicity assessments to elaborate safe pharmaceutical products (Heyerdahl-Viau et al., 2023).
4. Biotechnology for Consistency: To circumvent the challenges of field-collected material, the successful biosynthesis of key triterpenoids like lupeol in *V. encelioides* cell cultures offers a high-value pathway for the scale-up and standardization of non-toxic support molecules (Cerda-García-Rojas et al., 2000). This difficulty has prompted translational research into novel process engineering. A specific process patent demonstrates the development of an optimized hexane extraction method for *V. persicifolia* that dramatically increases the yield and purity of the lead eudesmane 4CDE (up to 3 g/kg of fresh material), successfully solving the scalability challenge for this high-value scaffold (Jimenez and Vazquez-Candanedo, 2015).

### 7.3 Translational roadblocks: bioavailability, stability, and scalability

Despite the high Selectivity Index (SI) of leads like the Arbusculin derivative, several translational challenges remain. First, the inherent lipophilicity of eudesmane cinnamates often results in poor aqueous bioavailability, necessitating advanced drug delivery systems or nano-formulations. Second, the off-target cytotoxicity observed in compounds with a “naked” α-methylene-γ-lactone moiety underscores the risk of systemic Michael-type additions with non-target proteins. Furthermore, chemical stability is a concern, as the relative percentage of key sesquiterpenes can fluctuate significantly depending on the plant’s maturity, complicating standardization. Finally, scalability remains a hurdle for scarce metabolites; however, current advances in cell culture biosynthesis and optimized extraction methods (yielding up to 3 g/kg of lead compounds) are beginning to address these industrial concerns.

While the compiled literature across independent laboratories demonstrates robust, reproducible *in vitro* cytotoxic and antimicrobial activities for *Verbesina* specialized metabolites, several fundamental translational limitations must be explicitly addressed. Mechanistic assays in cell culture evaluate direct target engagement under static conditions, completely bypassing host-specific pharmacokinetic (PK) and pharmacodynamic (PD) constraints. Highly reactive electrophilic scaffolds, such as the α-methylene-γ-lactone core characteristic of eudesmane structures, are prone to rapid systemic detoxification, off-target serum protein alkylation, or low metabolic stability before reaching therapeutic concentrations *in vivo*. Furthermore, high *in vitro* potency (IC_50_ or MIC) against naked cell membranes or unshielded pathogens does not guarantee systemic bioavailability or tissue-specific accumulation in complex living models. Consequently, until rigorous *in vivo* PK/PD profiles, acute/chronic systemic toxicity parameters, and animal disease models are systematically executed, the reported biological activities must be strictly interpreted as preliminary *in vitro* characterization rather than definitive therapeutic efficacy.

Beyond these pharmacokinetic hurdles, a transparent evaluation of the compiled *Verbesina* literature reveals clear methodological limitations that necessitate a cautious interpretation of current performance metrics. First, the quantitative SI values detailed across this review are compiled from highly heterogeneous baseline assays, utilizing diverse mammalian host controls and distinct tumor cell lines that vary significantly in susceptibility. Consequently, executing direct, head-to-head comparative evaluations across independent historical studies is fundamentally constrained by differing extraction strategies, localized solvent polarities, and divergent laboratory-specific methodologies. Finally, true *in vivo* safety indices, systemic clearance profiles, and deep pharmacokinetics are largely absent from the peer-reviewed literature for these specific eudesmane and *Arbusculin* templates. Embracing these constraints is vital for maintaining scientific transparency, signaling that while these specialized natural scaffolds manifest compelling *in vitro* anti-resistance potential, they require standardized, synchronized cross-laboratory validation before advancing further into preclinical pipelines.

### 7.4 Cross-class comparative benchmark and translational thresholds

To objectively position the *Verbesina* chemical library within the wider landscape of contemporary drug discovery, its baseline parameters must be systematically calibrated against established clinical standards and mature SL drug paradigms. Standard clinical anti-infectives (such as fluoroquinolones, macrolides, or lipopeptides like daptomycin) typically operate within highly optimized, narrow nanomolar to low micromolar minimum inhibitory concentration bounds (MIC ≤1.0 μg/mL), achieving exceptional host safety windows that frequently yield Selectivity Indices stretching into the hundreds (SI > 100). Conversely, standard small-molecule chemotherapeutic agents (such as doxorubicin or paclitaxel) showcase intense, sub-micromolar cytotoxic potency (IC_50_ < 1.0 μM) against proliferative cell lines, but are intrinsically limited by narrow safety margins when evaluated against healthy somatic tissues.

When *Verbesina*-derived SLs are evaluated against these benchmarks, their biological profiles reflect a distinct therapeutic modality. The genus’s high-potency antimicrobials, such as 6β-cinnamoyloxy-3β,4α-dihydroxyeudesmane, achieve impressive, low-micromolar inhibition metrics (MIC_100_ = 3.9 μg/mL) that are highly competitive with crude natural product discovery leads. Mechanistically, these bicyclic eudesmane matrices differ fundamentally from conventional anti-infectives; rather than selectively binding to singular, rapidly mutating bacterial enzymes, they leverage a dual-mode attack vector combining mechanical membrane disruption with target alkylation, preventing standard cross-resistance pathways. This structural approach mirrors the broader therapeutic archetype of clinically mature sesquiterpene lactones, most notably *Artemisinin* and its semi-synthetic analogues (such as artesunate). While *Artemisinin* relies on an iron-activated endoperoxide bridge to generate toxic free radicals inside localized pathogens, *Verbesina* templates exploit the spatial coordination of the cinnamoyloxy anchor to pre-align an α-methylene-γ-lactone core or induce specific bioenergetic mitochondrial uncoupling.

To guide future technology transfer and pipeline development, the co-occurring scaffolds of the genus can be filtered through strict quantitative translational gates:

1. High-Priority Translatable Scaffolds (SI ≥ 10 to > 20): Scaffolds that successfully decouple target eradication from host cytotoxicity fall into this viable category. The premier representative is the *Arbusculin* derivative isolated from *V. lanata*, which exhibits an outstanding anti-protozoal SI of up to 88.2, easily eclipsing the clinical safety window of the synthetic standard meglumine antimoniate (SI = 40.0). Similarly, highly functionalized cinnamoyloxy-eudesmane structures that manifest selective, low-micromolar antiproliferative trajectories (such as *Verbesindiol* cores targeting advanced prostate cancer lines at IC_50_ = 4.0 μM) satisfy the minimum thresholds required for advanced semi-synthetic optimization and structural modifications. These scaffolds represent robust chemical probes with a high likelihood of successful drug translation.
2. Untranslatable Toxicological Scaffolds (SI ≈ 1): Conversely, chemical entities that exhibit unshielded, highly electrophilic reactivity without cell-line specificity represent serious translational dead-ends. Simple, truncated germacrene lactones that display matching micromolar metrics across both microbial targets and non-cancerous human mesothelial models (GI_50_ ≈ 32.3 μM) offer zero therapeutic window. Furthermore, non-SL co-occurring toxins, most notably the acutely lethal guanidine alkaloid *galegine* (IC_50_ = 10.9 μg/mL), possess narrow safety margins that actively restrict extract-level utility. These structures are untranslatable into viable lead candidates and must be rigorously filtered out through bioassay-guided isolation to protect the empirical validity of the anti-resistance pipeline.

## 8 Conclusion

The *Verbesina* genus is a verified repository of complex, evolved chemical defenses, offering structurally unique sesquiterpenoid scaffolds with proven dual cytotoxic and antimicrobial activities. By identifying lead compounds with a favorable Selectivity Index (SI), and leveraging modern chemical synthesis and rigorous standardization protocols, the potential of this rich Mexican medicinal resource can be fully unlocked. The future of anti-AMR drug discovery is complex, but the multifaceted chemical mechanisms and translational feasibility demonstrated by *Verbesina* metabolites position them as promising next-generation scaffolds in the fight against antibiotic resistance.
