# Supplementary material for: Phytochemical diversity and therapeutic potential of Verbesina metabolites: a critical review of sesquiterpene lactones and translational feasibility
Source: Front Pharmacol. 2026 Aug 28;17:1899545. doi: 10.3389/fphar.2026.1899545 (PMC13563513; doi:10.3389/fphar.2026.1899545)
Supplement: Supplementary file 1 [file DataSheet2.pdf]

No.	Reference	Study type	Species	Voucher	Plant part	Extract / Fraction	Extraction method	Chemical characterization	Analytical techniques	Major compounds identified	Biological activity	Main findings	Methodological limitations	ConPhyMP quality
1	Gardner et al., 1961	Phytochemical isolation	*Verbesina virginica* L.	✕	Root	Petroleum ether extract	Continuous petroleum ether extraction	✓	UV, IR, optical rotation	α- and β-Verbesinol	NA	First isolation of verbesinol derivatives	No voucher; no fingerprint; no quantitative analysis	Moderate
2	Box et al., 1971	Phytochemical isolation	*Verbesina rupestris* (Urb.) Blake	✕	NR	Organic extract	Solvent extraction + chromatography	✓	UV, IR, NMR, MS, CD	Rupestrol derivatives	NA	New sesquiterpenes characterized	No voucher; plant part not reported; no fingerprint	Moderate
3	Wagner et al., 1974	Phytochemical isolation	*Verbesina myriocephala* Sch.Bip.	✓	Aerial parts	Methanolic extract	Methanol extraction + column chromatography	✓	UV, IR, NMR, MS	Rhamnocitrin glucuronide	NA	First flavonoid glucuronide isolated	No fingerprint; no metabolite quantification	High
4	Box & Chan, 1975	Phytochemical isolation	*Verbesina rupestris*	✓	Leaves, twigs	Acetone extract	Solvent extraction + chromatography	✓	IR, MS	Sesquiterpenes	NA	New terpenoids identified	No fingerprint	High
5	Bohlmann & Zdero, 1976	Comparative phytochemistry	Several *Verbesina* spp.	✓	Roots / aerial parts	Ether extract	Ether extraction + silica gel chromatography	✓	IR, NMR, MS	Eudesmane derivatives	NA	Comparative phytochemical profile	No fingerprint; no quantitative analysis	High
6	Box et al., 1977	Phytochemical isolation	*Verbesina rupestris*	✓	Leaves, twigs	Methanolic extract	Methanol extraction + chromatography	✓	UV, IR, NMR, MS, CD	Enantio-eudesmane sesquiterpenes	NA	Stereochemistry established	No fingerprint	High
7	Bohlmann & Lonitz, 1978	Comparative phytochemistry	Several *Verbesina* spp.	✓	Roots / aerial parts	Ether extract	Ether extraction + silica gel chromatography	✓	IR, NMR, MS	Eudesmane derivatives	NA	Comparative chemistry of seven species	No quantitative profile	High
8	Bohlmann et al., 1980	Comparative phytochemistry	Several *Verbesina* spp.	✓	Roots / aerial parts	Ether extract	Ether extraction + silica gel chromatography	✓	UV, IR, NMR, MS	Eudesmane sesquiterpenes	NA	Several new metabolites described	No fingerprint; no extraction yield	High
9	Glennie & Jain, 1980	Phytochemical isolation	*Verbesina encelioides*	✓	Flowers, leaves	70% ethanol extract	Ethanol extraction	✓	UV, paper chromatography	Flavonol diglycosides	NA	Flavonoid glycosides identified	No fingerprint	High
10	Oelrichs et al., 1981	Bioactivity-guided isolation	*Verbesina encelioides*	✓	Whole plant	MeOH/H_2O extract	Hydroalcoholic extraction + partition	✓	NMR, MS, TLC	Galegine	Hypotensive, toxicity	Isolation of galegine	No fingerprint; no metabolite quantification	High
11	Herz & Kumar, 1981	Phytochemical isolation	Several *Verbesina* spp.	✓	Aerial parts	$CHCl_3$ extract	Solvent extraction + chromatography	✓	IR, UV, NMR, MS	Verbesindiol	NA	Structure elucidation	No fingerprint	High
12	Herz et al., 1982	Structural confirmation	*Verbesina virginica*	NA	NA	Purified compound	NA	✓	X-ray crystallography	Verbesindiol	NA	Absolute configuration confirmed	Not an extract study	NA
13	Bohlmann et al., 1982	Phytochemical isolation	*Verbesina sordescens*	✓	Roots / aerial parts	Ether extract	Ether extraction + silica gel chromatography	✓	IR, MS, NMR	Eudesmane cinnamates	NA	Two new eudesmane derivatives	No fingerprint	High
14	Eichholzer et al., 1982	Bioactivity-guided isolation	*Verbesina encelioides*	✓	Aerial parts	MeOH/H_2O extract	Hydroalcoholic extraction	✓	NMR, MS	Galegine derivatives	Toxicity	Bioactive guanidine derivatives isolated	No fingerprint	High
15	Martínez et al., 1983	Phytochemical isolation	*Verbesina virgata*	✓	Aerial parts	$CHCl_3$ extract	Solvent extraction + chromatography	✓	IR, UV, NMR, MS, X-ray	Eudesmane triols	NA	New sesquiterpenes identified	No quantitative analysis	High
16	Ortega et al., 1985	Phytochemical isolation	*Verbesina seattonii*	✓	Aerial parts	$CHCl_3$ extract	Solvent extraction + chromatography	✓	IR, MS, NMR, X-ray	Elemanolides	NA	New elemanolides isolated	No fingerprint	High
17	Salmón et al., 1985	Phytochemical isolation	*Verbesina sphaerocephala*	✓	Leaves	$CHCl_3$ extract	Solvent extraction + preparative TLC	✓	UV, IR, NMR, X-ray	Verocephol	NA	Novel sesquiterpene γ-lactol	No fingerprint	High
18	Banerjee et al., 1985	Phytochemical isolation	*Verbesina eggersii*	✓	Aerial parts	Organic extract	Silica gel chromatography	✓	NMR, MS	Rearranged eudesmane derivatives	NA	New verbesindiol derivatives	No fingerprint	High
19	Jakupović et al., 1987	Comparative phytochemistry	Several *Verbesina* spp.	✓	Roots / aerial parts	Organic extracts	Solvent extraction + chromatography	✓	NMR, MS, IR	Sesquiterpenes	NA	Additional eudesmane derivatives	No fingerprint	High
20	Delle Monache et al., 1992	Bioactivity-guided isolation	*Verbesina caracasana*	✓	Whole plant	Methanolic extract	Methanol extraction + silica gel chromatography	✓	NMR, MS	Caracasanamide	Hypotensive	First isolation of caracasanamide	No fingerprint	High
21	Delle Monache et al., 1993	Pharmacological study	*Verbesina caracasana*	✓	Leaves	Methanolic extract	Soxhlet extraction	✓	UV, IR, HRMS, NMR	Caracasanamide	Hypotensive	Pharmacological characterization	No marker quantification	High

22	Jain et al., 2008	Phytochemical isolation + in vitro culture	*Verbesina encelioides*	✓	Seeds, callus	Ethanolic extract	Soxhlet extraction	✓	TLC, IR, NMR	Friedelin, epifriedelinol, lupeol, α-/β-amyrin, stigmasterol, betulin, β-sitosterol	Antibacterial, antifungal, antioxidant	Callus cultures produced the same major metabolites as the plant	No HPLC/GC-MS quantification	Moderate–High
23	Compagnone et al., 2008	Bioactivity-guided isolation	*Verbesina peraffinis*	✓	Leaves	Methanolic extract	Methanol extraction + chromatography	✓	HRMS, IR, NMR	Novel guanidine alkaloids, galegine	Hypotensive	Two new guanidine alkaloids identified	No quantitative analysis	High
24	Xu et al., 2009	Phytochemical isolation	*Verbesina virginica*	✓	Whole plant	Methanolic extract	Chromatographic fractionation	✓	HRMS, NMR, HPLC	Verbesinosides A–F	NA	Six new triterpenoid saponins isolated	No bioactivity	High
25	Xu et al., 2010	Phytochemical isolation	*Verbesina virginica*	✓	Whole plant	Methanolic extract	Chromatographic fractionation	✓	HRMS, NMR	Two new eudesmane derivatives	NA	New sesquiterpenes identified	No bioactivity	High
26	Abbas et al., 2016	Phytochemical isolation + pharmacology	*Verbesina encelioides*	✓	Aerial parts	Hydroalcoholic extract	Liquid–liquid partition + column chromatography	✓	UV, IR, MS, NMR	Taraxasterol acetate, β-amyrin, β-sitosterol, stigmasterol, quercetin glycoside, galegine, etc.	Cytotoxic, antibacterial, antifungal	Comprehensive phytochemical and biological characterization	No chromatographic fingerprint; no metabolite quantification	High
27	Van Nuland et al., 2013	Ecological study (pollination ecology)	*Verbesina alternifolia* (L.) Britton ex Kearney	✕	Whole plant (field populations)	NA	NA	✕	Field ecological surveys; pollinator visitation counts; vegetation density measurements	NA	Pollinator visitation	Prescribed fire increased *V. alternifolia* density by 44% and floral visitation by 54%, suggesting fire enhances pollination services through increased plant density.	No phytochemical data; no voucher reported; no chemical characterization	NA
28	Van Auken & Leonard, 2016	Ecological study (habitat ecology)	*Verbesina virginica* L.	✕	Whole plant	NA	NA	✕	Factorial field experiment (canopy × water × neighbors); survival and biomass measurements	NA	Ecological adaptation	Survival and biomass were greatest under canopy conditions without neighboring vegetation, demonstrating that competition and light availability largely determine the ecological niche of *V. virginica* .	No voucher reported; no phytochemical characterization	NA
29	Mora et al., 2015	Essential oil characterization	*Verbesina negrensis* Steyerm.	✓	Leaves	Essential oil	Hydrodistillation	✓	GC-MS	α-Pinene, α-humulene, δ-cadinene, limonene, bicyclogermacrene	Antibacterial	First characterization of the essential oil of *V. negrensis* , showing antibacterial activity against *Staphylococcus aureus* and *Enterococcus faecalis* .	No metabolite quantification beyond GC-MS composition; no fingerprint validation of plant extract	High
30	Pergomet et al., 2017	Synthetic chemistry (total synthesis of a natural product)	*Verbesina luetzelburgii*	✓ (natural product previously reported from authenticated material)	Not applicable (synthetic study)	Synthetic benzofuran derivative (5-(1-hydroxy-1-ethyl)-2-isopropylidene-2H-benzofuran-3-one)	Total chemical synthesis (8 steps from 4'-hydroxyacetophenone)	✓	NMR (^1H, ^13C), HRMS, IR, optical rotation, ECD, chiral HPLC	5-(1-Hydroxy-1-ethyl)-2 isopropylidene-2H-benzofuran-3-one	Synthetic methodology	First total synthesis of the only known benzofuran-3-one isolated from *V. luetzelburgii* , providing an efficient synthetic route (19–33% overall yield) that enables future biological evaluation and analogue synthesis.	No pharmacological evaluation; no phytochemical investigation of new plant material because this is a synthetic study	NA
31	Yaseen et al., 2017	Pharmacological screening	*Verbesina oerstediana*	✓	Bark; leaves	Crude acetone extracts	Soxhlet extraction (4 h)	✕	In vitro antimicrobial assays; neutrophil extracellular trap (NET) assay; confocal microscopy	Not identified	Immunomodulatory; enhancement of innate immune response against *Staphylococcus aureus*	Bark acetone extract enhanced the antimicrobial activity of human neutrophils and monocytes by stimulating neutrophil extracellular trap (NET) formation without direct antibacterial activity or cytotoxicity. Active metabolites remain unidentified.	Crude extract only; no phytochemical characterization or compound isolation; active constituents not identified	Low
32	Bezerra et al., 2018	Anatomical and phytochemical study	*Verbesina macrophylla*	✓	Leaves	Essential oil	Hydrodistillation (Clevenger, 3 h)	✓	Histochemistry, light microscopy, SEM, TEM, GC-FID, GC-MS	Germacrene D (37.3%), Germacrene D-4-ol (17.0%), β-caryophyllene (5.9%), bicyclogermacrene (5.3%), α-cadinol (4.5%)	Not evaluated experimentally	Described the ontogeny and ultrastructure of schizogenous secretory ducts, identified lipids, terpenes, alkaloids and mucopolysaccharides in the secretory system, and demonstrated that the essential oil is dominated by sesquiterpenes, especially germacrene D and its derivatives.	No pharmacological assays or bioactivity validation; chemical profile limited to essential oil	**Moderate**
**33**	Selener et al., 2019	Pharmacological screening	*Verbesina subcordata* DC.	✓ (BAF 767)	Aerial parts	Dichloromethane and methanol extracts	Sequential extraction with dichloromethane followed by methanol (24 h each, room temperature)	◐	TLC and HPLC (performed only on the most active extracts of other species)	Not identified for *V. subcordata*	Anti-*Trypanosoma cruzi* activity	Dichloromethane extract showed strong inhibition of *T. cruzi* epimastigotes (84.7% at 100 µg/mL), suggesting the presence of bioactive non-polar constituents.	No phytochemical characterization of *V. subcordata* extract; active compounds not isolated; no IC$_{50}$ or selectivity index	**Moderate**

**34**	Verma et al., 2019	Phytochemical study and pharmacological evaluation	*Verbesina encelioides* (Cav.) Benth. & Hook. f. ex Gray	✕	Flowers	Methanolic extract enriched in saponins	Extraction with 50% methanol followed by n-butanol partition	✓	TLC, HPLC, FTIR, UV-Vis spectroscopy, SEM, zeta potential analysis	Saponin-rich fraction (individual compounds not structurally elucidated)	Antioxidant; antibacterial against *Cutibacterium acnes* ; green synthesis of silver nanoparticles	Saponin-rich extracts exhibited antioxidant activity and enabled biosynthesis of silver nanoparticles with antibacterial activity against acne-associated bacteria.	No voucher specimen; no LC-MS or NMR characterization; compounds not structurally identified	**Moderate–Low**
35	Farshori et al., 2021	Pharmacological study	*Verbesina encelioides*	✓	Aerial parts	Ethanolic and aqueous extracts	Maceration	✕	MTT, ROS assay, RT-qPCR	Not identified	Anticancer	Ethanolic extract induced ROS-mediated apoptosis in HCT-116 cells.	No chemical fingerprint; no metabolite identification; crude extracts only.	Moderate-Low
36	de Veras et al., 2021	Essential oil characterization	*Verbesina macrophylla*	✓	Leaves	Essential oil	Hydrodistillation	✓	GC-MS, GC-FID	Germacrene D, Germacrene D-4-ol, Bicyclogermacrene, (E)-Caryophyllene	Antimicrobial; anti-inflammatory; antipyretic	Essential oil exhibited antimicrobial, anti-inflammatory and antipyretic activities with low acute toxicity.	No bioassay-guided isolation; mechanisms not investigated.	High
37	Velasco-Ramírez et al., 2022	Agricultural / biostimulant study	*Verbesina sphaerocephala*, *V. fastigiata*	✓	Leaves	Aqueous extract	Aqueous extraction (autoclaved)	Partial	Multiparameter photometer (pH, EC, NH_4NO_3, Ca, Mg, PO_4^{3-}); no metabolomic profiling	Not identified	Plant growth promotion	Aqueous extracts promoted vegetative and root development of *Fragaria × ananassa* , improving several agronomic variables compared with the control.	No metabolite identification; no chromatographic fingerprint; no bioassay-guided fractionation; agricultural study only.	Moderate-Low
38	Velasco-Ramírez et al., 2022	Agricultural / allelopathy and biostimulant study	*Verbesina sphaerocephala*, *V. fastigiata*	✓	Leaves	Aqueous extract	Aqueous extraction (autoclaved)	Partial	Multiparameter photometer; agronomic measurements	Not identified	Allelopathic and plant growth-promoting	Extracts reduced seed germination but enhanced seedling growth of *Solanum lycopersicum* and *Cucumis sativus* under selected conditions, suggesting concentration-dependent allelopathic and biostimulant effects.	No chromatographic fingerprint; no identification of bioactive metabolites; no isolation of active compounds.	Moderate-Low
39	Kaur et al., 2022	Systematic review	*Verbesina encelioides*	NA	NA	NA	NA	Review	Literature review	Summarizes flavonoids, terpenoids, alkaloids, phenolics, sterols, triterpenoids, galegine and related metabolites reported in previous studies	Review of pharmacological and biological activities	Comprehensive review covering phytochemistry, ethnomedicine, allelopathy, toxicity, antimicrobial, anticancer, antiprotozoal and hypoglycemic activities.	Secondary source; no original experimental data; no standardized phytochemical analysis.	NA
40	Lahlali et al., 2023	Experimental bioassay	*Verbesina encelioides*	✕	Aerial parts	Aqueous extract	Maceration in water	Partial	Total phenolics, total flavonoids, cyanobacterial bioassays	Phenolics and flavonoids quantified; individual compounds not identified	Anti-cyanobacterial	Aqueous extract strongly inhibited *Microcystis aeruginosa* growth in a concentration-dependent manner and reduced chlorophyll and carotenoid contents.	No chromatographic fingerprint; no identification of individual allelochemicals; no bioassay-guided isolation.	Moderate
41	Heyerdahl-Viau et al., 2023	Narrative review	*Verbesina crocata*	NA	Mainly aerial parts (reviewed studies)	Methanolic and aqueous extracts (compiled from literature)	Various (compiled from published studies)	Partial	Literature review of phytochemical and pharmacological studies	Terpenes/steroids, glycosides, alkaloids, coumarins, quinones, saponins, tannins; daucosterol, lupeol, lupeol acetate, and galegine reported as putative bioactive constituents	Review of wound healing, antidiabetic and diuretic activities; ethnopharmacological uses	Comprehensive synthesis of the available evidence on *V. crocata* , concluding that scientific support is currently limited but experimental studies support wound-healing, antidiabetic and diuretic activities. The review emphasizes the need for phytochemical characterization, toxicity evaluation and mechanistic studies.	Secondary source; no original experimental data; phytochemical evidence remains incomplete because most primary studies lacked metabolite identification and chromatographic fingerprinting.	NA
42	Bezerra et al., 2024	Original research	*Verbesina macrophylla*	Yes (Herbarium UESC No. 19101)	Leaves and inflorescences	Aqueous extracts	Maceration in demineralized water (24 h) followed by filtration and lyophilization	No (extract not chemically profiled)	SEM, optical microscopy, fluorescence microscopy (Sytox Green), TEM, biological assays	None identified experimentally (authors discuss sesquiterpene lactones based on literature but they were not analyzed in this study)	Antifungal and trypanocidal	Inflorescence extract inhibited *Candida tropicalis* (~41%) by disrupting plasma membrane integrity. Both leaf and inflorescence extracts showed dose- and time-dependent trypanocidal activity against *Trypanosoma cruzi* , reaching complete elimination at 200 µg/mL, accompanied by ultrastructural alterations.	No chromatographic characterization (HPLC/LC-MS/GC-MS); active compounds were not isolated; only crude aqueous extracts evaluated; no toxicity toward mammalian cells.	**Low**

43	Mehal et al., 2023	Ecological study	*Verbesina encelioides*	Not applicable	Whole plant (field populations)	None	Not applicable	No	None	None	Ecological invasion	Evaluated the ecological impact of the invasive species *V. encelioides* on native vegetation across dryland ecosystems in India. The invasion significantly reduced species diversity and altered vegetation composition, especially in arid and semi-arid regions. The study discusses ecological mechanisms and cites previously reported allelochemicals but performs no phytochemical analyses.	Not a pharmacological or phytochemical study; no extraction, metabolite identification or biological assays on plant extracts.	**NA**
44	Ramírez-Herrera et al., 2025	Original research	*Verbesina crocata*	No reportado	Leaves	Aqueous extract	Decoction (70 °C, 10 min)	**Qualitative only**	Phytochemical screening, UV-Vis, FTIR, XRD, SEM-EDS	Alkaloids, tannins, saponins, phenolics, terpenoids and flavonoids (qualitative only)	Green synthesis of Ag/AgCl nanoparticles ; phytotoxicity	The aqueous extract successfully synthesized Ag/AgCl nanoparticles. Nanoparticles showed concentration-dependent phytotoxicity in *Raphanus sativus* . Extract alone also exhibited allelopathic effects.	No chromatographic profiling (HPLC, LC-MS or GC-MS); metabolites not identified individually; no quantitative phytochemical analysis.	**Low**
45	Rodríguez-Valdovinos et al., 2024	Original research	*Verbesina sphaerocephala*	Not reported	Stems	Aqueous extract and ethyl acetate/methanol fractions	Aqueous extraction followed by liquid-liquid partition	**Yes**	UPLC-MS, antioxidant assays (DPPH, ABTS, FRAP, TAC), antifungal assays	Protocatechuic acid, vanillic acid, hydroxybenzoic acid, rutin, vanillin, chlorogenic acid, quercetin, kaempferol and other phenolics	Antioxidant and antifungal	The aqueous extract and especially the ethyl acetate fraction exhibited high antioxidant capacity and strong inhibition of *Botrytis cinerea* . UPLC-MS identified 12 phenolics in the crude extract and 18 in the ethyl acetate fraction.	Only aqueous extracts evaluated; no isolation of active constituents; no in vivo validation.	**High**
46	Hernández-Pérez et al., 2025	Original research	*Verbesina sphaerocephala*	Not reported	Leaves	Aqueous extract	Infusion	Partial	Colorimetric phytochemical assays, FTIR	Phenolics, flavonoids, phenolic acids, tannins, alkaloids, proanthocyanidins, anthocyanins	Biostimulant (plant growth promotion)	Foliar + substrate application (0.75 g/L) significantly improved tomato growth, flowering and yield under greenhouse conditions, suggesting a biostimulant effect associated with phenolic metabolites.	No chromatographic identification (HPLC/LC-MS); study focused on agronomic performance rather than pharmacological activity.	**Moderate**
